# Towards harmonisation of testing of nanomaterials for EU regulatory requirements on chemical safety – A proposal for further actions

**DOI:** 10.1016/j.yrtph.2023.105360

**Published:** 2023-03

**Authors:** Eric A.J. Bleeker, Elmer Swart, Hedwig Braakhuis, María Luisa Fernández Cruz, Steffi Friedrichs, Ilse Gosens, Frank Herzberg, Keld Alstrup Jensen, Frank von der Kammer, Jolinde A.B. Kettelarij, Jose María Navas, Kirsten Rasmussen, Kathrin Schwirn, Maaike Visser

**Affiliations:** a National Institute for Public Health and the Environment (RIVM), P.O. Box 1, 3720 BA, Bilthoven, the Netherlands; b Instituto Nacional de Investigación y Tecnología Agraria y Alimentaria (INIA), CSIC, Ctra. De la Coruña Km 7,5, 28040, Madrid, Spain; c AcumenIST SRL, Rue Fétis 19, 1040, Etterbeek, Belgium; d German Federal Institute for Risk Assessment (BfR), Max-Dohrn-Str. 8-10, 10589, Berlin, Germany; e The National Research Centre for the Working Environment (NRCWE), 105 Lersø Parkallé, DK-2100, Copenhagen, Denmark; f University of Vienna, Centre for Microbiology and Environmental Systems Science, Department of Environmental Geosciences, Josef-Holaubek-Platz 2, 1090, Vienna, Austria; g European Commission, Joint Research Centre (JRC), Ispra, Italy; h German Environment Agency (UBA), Woerlitzer Platz 1, 06844, Dessau-Rosslau, Germany

**Keywords:** Nanomaterials, Information requirements, Guidance, Test guidelines, Nanosafety

## Abstract

Over the recent years, EU chemicals legislation, guidance and test guidelines have been developed or adapted for nanomaterials to facilitate safe use of nanomaterials. This paper provides an overview of the information requirements across different EU regulatory areas. For each information requirement, a group of 22 experts identified potential needs for further action to accommodate guidance and test guidelines to nanomaterials. Eleven different needs for action were identified, capturing twenty-two information requirements that are specific to nanomaterials and relevant to multiple regulatory areas. These were further reduced to three overarching issues: 1) resolve issues around nanomaterial dispersion stability and dosing in toxicity testing, in particular for human health endpoints, 2) further develop tests or guidance on degradation and transformation of organic nanomaterials or nanomaterials with organic components, and 3) further develop tests and guidance to measure (a)cellular reactivity of nanomaterials. Efforts towards addressing these issues will result in better fit-for-purpose test methods for (EU) regulatory compliance. Moreover, it secures validity of hazard and risk assessments of nanomaterials. The results of the study accentuate the need for a structural process of identification of information needs and knowledge generation, preferably as part of risk governance and closely connected to technological innovation policy.

## Highlights

• An overview is provided of information requirements for nanomaterials in the EU.
• Needs for further action to address nanospecific issues are identified.
• Priority needs to adapt or develop TGs/GDs are identified.
• This works contributes to better fit-for-purpose methods for regulatory compliance.

## List of abbreviations

AOP: Adverse outcome pathways
ECHA: European Chemicals Agency
EFSA: European Food Safety Authority
EMA: European Medicines Agency
EU H2020: EU's research and innovation funding programme in 2014–2020
GDs: Guidance Documents (OECD)
IATA/ITS: Integrated Approaches to Testing and Assessment/Intelligent Testing Strategy
ISO: International Organization for Standardization
NAM: New Approach Methodologies
NMEG: ECHA Nanomaterials Expert group
MAD: Mutual Acceptance of Data
OECD: Organisation for Economic Co-Operation and Development
REACH: Registration, Evaluation, Authorisation and Restriction of Chemicals (EU)
REFINE: Regulatory Science Framework for Nano(bio)material-based Medical Products and Devices
SCCS: Scientific Committee on Consumer Safety
SCENIHR: Scientific Committee on Emerging and Newly Identified Health Risks
TGs: Test Guidelines (OECD)
TGP: Test Guidelines Programme (OECD)
TSCA: Toxic Substance Control Act (USA)
WNT: Working Party of National Coordinators of the Test Guidelines Programme (OECD)
WPMN: Working Party on Manufactured Nanomaterials (OECD)

## 1 Introduction

In the last two decades, knowledge on the potential health, safety and environmental impacts of nanomaterials has grown considerably. For example, major progress in understanding nanomaterials has been made in the OECD testing programme of manufactured nanomaterials (OECD, 2015) and in the many European projects in the NanoSafety Cluster (NanoSafety Cluster, 2007). The progress in gained knowledge and understanding of nanomaterials is illustrated by the increase in the number of publications on hazards and risks of nanomaterials (e.g. Roco, 2018; Zhu et al., 2020). Research underlined that many properties of nanomaterials differ from those of conventional chemicals, and that some properties of conventional chemicals are not relevant for most nanomaterials (e.g. log *K*_OW_). Nevertheless, in hindsight several authors identified shortcomings in the quality of the available nanotoxicological data, in particular those derived from the OECD testing programme (e.g. Hansen et al., 2017; Riediker et al., 2016). While these shortcomings limit direct applicability of these data for the hazard and risk assessment, the outcomes of the testing programme still represent an impressive amount of data through which insights were gained that helped to improve the risk assessment and management of nanomaterials (Riediker et al., 2016). For example, there is a consensus that, although nanomaterials generally fit within the existing regulatory frameworks, the specific properties of nanomaterials should be taken into account in their risk assessment. This may require adaptation of regulatory frameworks and the associated test methods to allow an adequate hazard and risk assessment. The OECD Council provided a recommendation on safety testing and assessment of manufactured nanomaterials that outlined this issue (OECD, 2013). As a result, activities were started to develop new or adjust existing OECD Test Guidelines (TGs) and Guidance Documents (GDs) to accommodate the specific properties of nanomaterials (e.g. Rasmussen et al., 2019). In parallel, legislation in different jurisdictions has been adapted to accommodate nanomaterials (e.g. the EU regulation REACH (EU, 2018), the EU regulation on cosmetics (EC, 2009b) the EU novel foods regulation (EC, 2015) and TSCA (US-EPA, 2017) in the USA).

Harmonised and standardised methods are essential to obtain the necessary information to address regulatory requirements, not only for nanomaterials, but for chemicals in general. The International Organization for Standardization (ISO, www.iso.org) and the OECD are the main globally operating organisations that provide such methods. ISO develops standards for any conceivable aspect of measuring and describing nanotechnologies, except regulatory methods. The OECD develops methods for regulatory testing. Therefore, much of the current chemical legislation strongly relies on OECD TGs to gain the necessary information for regulatory requirements. In addition, specific regulations (or accompanying guidance) may refer to other methods (e.g. from scientific literature), although this is generally limited to areas where harmonised or standardised methods are lacking. Within REACH legislation, OECD test methods are mandatory for regulatory compliance according to regulation EC 440/2008 (EC, 2008a).

The Mutual Acceptance of Data (MAD, www.oecd.org/chemicalsafety/testing/council-acts-on-mutual-acceptance-of-data.htm) is an important cornerstone of the use of OECD TGs. It avoids unnecessary duplication of tests and thereby unnecessary (animal) tests and costs. Furthermore, the use of harmonised and standardised methods can provide a strong basis for the knowledge building on hazards and risks, and as such, forms a cornerstone for implementing new paradigms such as safe-by-design and sustainable chemistry (EC, 2020).

In 2006, the Working Party on Manufactured Nanomaterials (WPMN) was established with a focus on nano-related OECD activities. As a subsidiary body of the OECD Chemicals Committee, the WPMN started by overseeing the OECD testing programme of manufactured nanomaterials (OECD, 2015). A preliminary WPMN review of the existing TGs was also conducted to identify potential needs for adaptation of test methodologies (OECD, 2009). These needs were further explored in several workshops, e.g. on physico-chemical properties (OECD, 2014b; OECD, 2016a), ecotoxicity and environmental fate (OECD, 2014a), genotoxicity (OECD, 2014c) and toxicokinetics (OECD, 2016b). Rasmussen et al. (2016) provides an overview of the first decade of work done in the WPMN. More recent activities can be found via the OECD website (www.oecd.org/env/ehs/nanosafety).

Over the last years, much of the ongoing work has focused on adapting existing and/or developing new OECD TGs and GDs for nanomaterials. To this end, closer links have been sought between OECD WPMN and the OECD Test Guidelines Programme (TGP) that is overseen by the Working Group of National Co-ordinators of the TGP (WNT). This resulted in a number of OECD documents, including TGs (OECD, 2017a; OECD, 2017b; OECD, 2017c), and GDs (OECD, 2018; OECD, 2020; OECD, 2021a; OECD, 2021c) with more being underway (Rasmussen et al., 2019). More details are provided below in an overview of OECD activities.

A large part of the current work in this area is supported by the so-called Malta Initiative,^2^ e.g. by ongoing European research projects like Gov4Nano (www.gov4nano.eu) and NanoHarmony (www.nanoharmony.eu). These projects provide a scientific basis for the development of a range of new and updated OECD TGs and GDs. Ongoing OECD activities and their progress towards the development and adaptation of TGs and GDs are summarised in the yearly updated WNT Workplan of the Test Guidelines Programme (OECD, 2022c). Recent examples include new TGs on nanomaterial particle size and size distribution of nanomaterials (OECD, 2022b) and on determination of the volume specific surface area of manufactured nanomaterials (OECD, 2022a), which were both approved by the WNT in April 2022.

The priority for development and adaptation of TGs and GDs within the Malta Initiative is largely tailored towards the testing needs under the EU REACH regulation on industrial chemicals (EU, 2018) and were initially identified by the European Chemicals Agency (ECHA) Nanomaterial Expert Group (NMEG).^3^ This raises the question of whether other (EU) regulatory areas (e.g. for specific substance applications) have specific needs for nanomaterials that have been overlooked in earlier assessments and that may require additional adaptations or developments of OECD TGs or GDs.

With this in mind, we present a comprehensive overview of the information requirements of the different European chemical regulatory areas. To our knowledge, this is the first time an overview is compiled of all areas in European legislation that explicitly address nanomaterials. Other authors have performed similar exercises but focused on a specific regulatory area (e.g. the medical application areas; Halamoda-Kenzaoui et al., 2019), or parts of such a regulatory area (e.g. environmental safety assessment under REACH; Nielsen et al., 2021). Using this overview, potential needs for further action to address nanospecific issues were identified by a group of nanosafety experts from various (European) research institutions and regulatory bodies. In the current paper we examine the information requirements and potential needs for a range of different areas (see details in the next section), including the (veterinary) medical application areas (i.e. (veterinary) medicinal products and medical devices). However, in the overall assessment and suggested prioritisation of needs for further action, the (veterinary) medical application areas received less attention (further explained below), although we acknowledge that these areas can support the prioritising of regulatory needs.

As explained above the different EU regulatory areas typically rely on OECD TGs and GDs for testing and guidance towards addressing regulatory requirements. Therefore, in this paper we focus on these OECD documents. We acknowledge that other standardised methods (e.g. ISO) may occasionally be used in various EU chemicals legislations, but these methods will not be the focal point of the current paper.

This paper 1) explains the approach used for the identification of information requirements across regulatory areas and the needs for further action to accommodate nanospecific issues, 2) gives an overview of the identified needs for further action, and 3) proposes prioritisation of further actions. In the outlook at the end of this paper, we reflect on the analysis and highlight broader perspectives and additional overarching needs to facilitate safe use and production of nanotechnology.

The overview presented here may help to identify and to prioritise further work on the development and adaptation of OECD TGs for nanomaterials applicable across multiple regulatory areas dealing with chemicals. Furthermore, it should be noted that while the overview summarises regulatory requirements for nanomaterials from an EU perspective, the identified key priority needs towards test methodologies are anticipated to have relevance in other (national) jurisdictions as well.

## 2 Methods for the identification of needs for further action

### 2.1 Identification of information requirements across regulatory areas

In a first step towards identifying further regulatory needs and prioritising future work on TGs, we expanded the initial overview of regulatory requirements that was used for the prioritisation conducted by ECHA-NMEG^4^ (i.e. those for REACH (EC, 2006; EU, 2018)) to legislation on specific substance applications. These comprise the food and feed sector (including plant protection products) (EC, 2015; EU, 2011; EU, 2013), cosmetics (EC, 2009b), biocides (EU, 2012), medical devices (EU, 2017), medicinal products for human use (EC, 2001), and veterinary medicinal products (EU, 2019). For each legislation, we focused on documents that specifically consider nanomaterials and/or their specificities (further details below). Note that for the identification of information requirements we considered legislation and guidance documents that were applicable in June 2021. As such, updates to information requirements released since are not included in the overview. With regard to (veterinary) medicinal products and medical devices, we focused on non-clinical requirements, as clinical requirements rely much less on OECD TGs than the other regulatory areas. Furthermore, the regulatory needs for nanomaterials in medicinal products and medical devices have recently been reviewed in detail elsewhere (Halamoda-Kenzaoui et al., 2019, 2021). Therefore, these areas receive less focus here. However, they may still support prioritising regulatory needs for (adapted) test methodologies.

Because some of the above EU legislation do not specify the requirements in detail, we additionally considered accompanying regulatory guidance documents, notes or reflection papers from ECHA, the Scientific Committee on Consumer Safety (SCCS), the European Food Safety Authority (EFSA), the European Medicines Agency (EMA), and the Scientific Committee on Emerging and Newly Identified Health Risks (SCENIHR)to gather the necessary information. Additionally, standards from the ISO can be relevant and have been considered in this paper. Table S1 in the Supplementary Information gives a full list of the documents that were considered in our analysis. It should be acknowledged that in some regulatory areas additional instruments are in place to ensure that potential effects, which are not captured in standard requirements, can still be addressed. In REACH, for instance, immunotoxicity can be addressed in a procedure of Substance Evaluation. However, in this document for the identification of REACH requirements, we only considered standard information requirements as outlined in REACH Annexes VI-X.

In addition to the legislation considered here, there is other EU legislation that deals with chemical safety such as Classification, Labelling and Packaging (CLP; EC, 2008b) or Chemical Agents Directive (CAD; EC, 1998). However, this legislation does not specifically ask for (toxicity) testing of chemicals (or nanomaterials) but essentially relies on existing data. Therefore, they were not considered in this overview.

It should be noted that some of these regulatory areas may use different definitions for the term ‘nanomaterial’, or indeed have no definition. This may affect the specific requirements for a given substance in a particular regulatory area. In compiling an overview of regulatory requirements, however, we ignored these differences for several reasons. The differences in definitions are less important for the information requirements and information generation and often relate to the specific requirements for the regulatory area. Furthermore, these definitions may be (further) harmonised as a result of the review and update of the EC recommendation (EC, 2022). Independent of a specific definition, the additional regulatory information requirements for nanomaterials generally relate to their particulate nature, i.e. small, solid and non-readily dissolving materials that behave differently and may induce toxicity via different or additional mechanisms compared to conventional chemicals for which legislation was originally designed. It should be noted that nanomaterials may comprise a variety of morphologies and forms, e.g. as 2D, 3D and complex or advanced materials. As the knowledge on the specific safety and regulatory issues for these later generations of nanoforms (e.g. multi-component or ‘advanced’ nanomaterials) is still in an early phase of development, the focus of this exercise was more on the first generation nanomaterials (i.e. as solid particles according to the 2022 recommendation (EC, 2022)). However, regulatory issues identified that relate to these more complex (nano)materials are not explicitly excluded.

By including information requirements from regulatory areas other than industrial chemicals (i.e. REACH), we greatly expanded the total number of identified regulatory requirements for chemicals in the EU. In many cases, different regulatory frameworks have similar requirements, although their exact descriptions of the information requirements often vary. Nevertheless, they may rely on the same standardised/harmonised test methods. To minimise redundancy, we merged comparable information requirements of different frameworks. For example, in the cosmetics regulations, information on “catalytic activity” is required, in the food/feed area “reactivity” information is required, and for medical devices the importance of “chemical reactivity/catalytic activity and photocatalytic activity” is highlighted. In the final overview, these cases were merged as “reactivity” as a single information requirement.

### 2.2 Analysis of needs for further action by RIVM experts

For each of the identified information requirements, seven experts from the National Institute for Public Health and the Environments (RIVM) in the Netherlands identified an initial list of potential issues in obtaining the required information for nanomaterials. For each of the identified issues, recent and ongoing activities were assessed to identify whether the issues are already addressed in OECD TGs/GDs or in ongoing projects that aim to adapt or develop such methods. This allowed the identification of where further work towards adaptation of existing or development of new OECD TGs or GDs is potentially needed. It should be noted that an identified potential need indicates that there is a need for harmonisation to improve comparability of results. An identified need should not be interpreted as a rejection of the applicability of an existing relevant OECD TG/GD for regulatory purposes. This is further discussed in the outlook section at the end of this paper.

### 2.3 Further analysis by EU experts on the needs for further work

In a next step, 15 experts from Europe working for (in total nine different) research organisations or (in total two different) EU regulatory bodies (hereafter, ‘EU experts’) were invited to 1) critically reflect on the analysis, 2) to identify omissions and 3) to discuss and find a consensus on the potential needs for further action. All of the 22 experts (7 experts from the RIVM and 15 invited experts from other European institutes) are experts on nanomaterial safety and were chosen so that knowledge on each of the seven regulatory areas would be represented (i.e. experts on environmental safety, human health, the (veterinary) medical application area, food and feed, cosmetics and biocides) and so that different geographical areas and levels of jurisdiction in Europe (Denmark, United Kingdom, Germany, Austria, Spain, the Netherlands and European Commission) were represented. Two of the fifteen EU experts work for a regulatory body and may be involved in the development of (regulatory) guidance for nanomaterials in the EU. The views of these experts may deviate from those of the regulatory body they work for, but we chose to include them in the discussions as their detailed understanding of development of nanomaterial regulations and guidance appeared essential. Further, the EU experts are all involved in multiple EU projects focussed on nanomaterials safety, risk assessment and governance and/or harmonisation of test methods (e.g. the development and adaptation of OECD TGs/GDs for nanomaterials).

By inviting a broad group of nanomaterials experts with different expertise and backgrounds, we aimed to test, refine and complement the RIVM analysis and to bring together the view of the EU nanomaterials safety and regulatory field. Specifically, the EU experts were asked to provide written input on the following two questions.

1. Can you agree on our initial evaluation of the (potential) need for further adaptation/‌development of OECD TGs/GDs?
2. Do you see major omissions in the regulatory requirements for the regulations assessed?

During July and August of 2021, in total 12 of the 15 EU experts provided in total eight sets of written feedback on the RIVM analysis, sometimes combining views from different experts from one institute in a single set. Three of the EU experts did not provide written feedback. The feedback was then collected in a single file and discussed further in an online meeting on the 3^rd^ of September 2021. This meeting, moderated by the RIVM, was attended by all EU experts who had provided written feedback. During the meeting the written feedback results were presented, discussed and clarified where needed. In these discussions, the needs for further action identified by RIVM were critically assessed and re-assessed as needed. Further, the needs were discussed with the goal of reaching a consensus on those information requirements for which different opinions were expressed in the written feedback. Each of these discussions resulted in a consensus without a formal (voting) procedure. This resulted in a final list of research needs for the different information requirements that was agreed amongst all the experts. After the meeting, a short summary of the discussions, accompanied by the consensus list of research needs and an annex of the detailed reflections for the different information requirements was shared with all the experts. All experts agreed with the content of these documents.

There was redundancy in the collected input (i.e. for some information requirements multiple experts provided similar inputs). Therefore, after the online meeting, the collected input was summarised so that only unique points of discussion would remain. The summaries of the input of the experts are provided in the Supplementary Information (Tables S3–S5). We then further condensed the expert input to improve the readability and to conform to the journal's publication standards. These condensed summaries are provided in Table 2 and Table 3. Given the substantial additions by the EU experts, all were invited to be author on this paper.

### 2.4 Information requirements for nanomaterials in EU chemical regulatory areas

The identification of similarities and differences in information requirements among different regulatory areas may help to identify priority research needs towards adaptations of TGs/GDs for nanomaterials and how these adaptations should be addressed. For instance, priority may increase when several regulations benefit from such adaptations.

To this end, we identified 136 information requirements in total among the seven considered EU chemical regulatory areas. Many of these are general requirements for chemicals that are also relevant for nanomaterials (e.g. toxicological endpoints do not differ, although testing for these endpoints may require nano-specific adaptations). These information requirements are summarised in Table 1. A detailed list of all the 136 identified information requirements is given in Supplementary Information Table S2. It should be noted that while the regulatory areas of industrial chemicals, cosmetics, biocides, medical devices and human and veterinary medicinal products each correspond to individual specific legislation, the area of food and feed as depicted in these tables comprises several sets of legislation along the food and feed chain, including plant protection products and novel foods.

**Table 1:** Overview of requirements for substances per EU regulatory area. All 136 identified information requirements are grouped within one of twenty information requirement categories. A comprehensive overview is provided in Supplementary Information Table S2. Numbers in brackets in the first column indicate the total number of different information requirements per category. Next columns indicate numbers per regulatory area. Hyphens (−) indicate that there is no information requirement for a given regulatory area. Numbers in bold indicate that all information requirements in that category are required for the specific regulatory area.

Information requirement category with in brackets the total number of identified requirements per category	EU regulatory area						
	REACH^a^	Cosmetics	Food and feed^b^	Biocides	Medicinal products for human use	Medical devices	Veterinary medicinal products
**Physico-chemical properties**							
Chemical name/identifier and molecular structural properties (9)	**9**	**9**	**9**	**9**	**9**	**9**	**9**
Composition and (im)purities (7)	**7**	**7**	**7**	**7**	**7**	**7**	**7**
Physical descriptors of nanoform/substance (e.g. size, shape, surface) and production methods (13)	9	11	12	9	9	12	6
Basic physical-chemical properties (e.g. melting/freezing/boiling point, vapor pressure, pH, K_OW_) (16)	11	15	10	**16**	12	8	14
Stability in relevant media (3)	1	**3**	**3**	2	2	2	1
Flammability and explosive properties (5)	**5**	3	1	**5**	–	2	–
Interaction with drugs and other active ingredients (3)	–	–	–	–	**3**	2	1
**Human health**							
Sensitisation, irritation and inflammation (10)	7	8	3	4	8	6	3
Cytotoxicity and reactivity (3)	–	2	**3**	1	–	2	2
Genotoxicity, mutagenicity, carcinogenicity (5)	**5**	4	**5**	**5**	**5**	**5**	**5**
Acute/short term toxicity (4)	**4**	1	**4**	**4**	1^c^	**4**	3
Long term toxicity (e.g. sub-chronic, reproduction) (6)	**6**	4	5	4	4	**6**	3
Uptake/kinetics (4)	1	3	1	2	2	1	2
Other requirements (endocrine disruption, neurotoxicity, immunotoxicity, microbiome interactions, use of data in humans, bioburden control and pharmacodynamics parameters) (7)	2	2	5	4	3	1	5
**Effects on biotic systems**							
Invertebrate toxicity (8)	6	–	**8**	7	3	–	**8**
Plants and algae toxicity (4)	3	–	3	3	3	–	3
Microbial toxicity (2)	**2**	–	1	**2**	**2**	–	1
Fish (toxicity and accumulation) (6)	**6**	–	4	**6**	3	–	4
Long term testing in birds or mammals (5)	1	–	3	3	–	–	–
**Environmental fate and behaviour**							
(A)biotic degradation (12)	9	–	**12**	11	5	–	8
Fate and behaviour in the environment (4)	**4**	–	**4**	**4**	**4**	–	**4**

a Note that requirements for REACH in this overview are limited to those mentioned in the Annexes VI-X in the REACH regulation (EC, 2006; EU, 2018).

b The food and feed regulatory area as depicted in this table includes several regulations along the food and feed chain, including PPPs and novel foods, see also the Supplementary information for a list of considered documents (Table S1).

c Acute toxicity is an information requirement within Directive 2001/83/EC on the Community code relating to medicinal products for human use (EC, 2001). However, since then it has been agreed to remove the guideline on single dose toxicity for medicinal products because the data obtained in single dose toxicity studies is considered to be of limited value and because information on acute toxicity can be obtained in other types of toxicity studies (EMA, 2010).

Below we provide a brief summary of similarities and differences in information requirements between regulatory areas for physico-chemical properties, human health related endpoints, and endpoints related to biotic systems and the environment.

#### 2.4.1 Physico-chemical properties

The overview shows that there are many similarities in information requirements between the different regulatory frameworks, especially for information on substance identification and descriptors (Table 1). For example, all regulatory areas require similar information on chemical identifiers, molecular structure, purity, composition of a (nanoform of a) substance and morphological characterisation (e.g. shape, size and surface properties of a nanomaterial), albeit sometimes only specified in guidance (e.g. EFSA Scientific Committee et al., 2021), because especially older legislation does not contain the string “nano” (e.g. EC, 2009a). Some differences appear when considering other physico-chemical properties. For example, in contrast to most areas, REACH does not require information on colour and pH, whereas food and feed legislation does not require information on e.g. odour and surface tension. For the (veterinary) medicinal products and medical devices legislation, information on surface tension and UV absorption is not required, which is in contrast to the information requirements in most other regulatory areas. Some of the differences in information requirements can be explained by the differences in the type of application or use of substances that are regulated within the different frameworks. For example, cosmetics, food and feed, and (veterinary) medicinal products and medical devices regulations do not require information on flammability and explosive properties. Information on the interaction of a substance with drugs and other active ingredients is only required in (veterinary) medicinal products and medicinal devices legislation.

#### 2.4.2 Human health

Among human health information requirements, all chemical regulatory areas in the EU have very similar information requirements on, for example, long-term toxicity (including reproductive toxicity), mutagenicity or carcinogenicity (Table 1). Differences in human health information requirements can be partly explained by differences in the application or uses of the chemicals regulated within the various regulatory areas. For example, in the legislation for cosmetics and the directive for medicinal products, requirements related to skin sensitisation and skin irritation are strongly expanded. Further, in cosmetics legislation, and in contrast to all other areas, *in vivo* studies are not an information requirement because conducting *in vivo* studies on vertebrate animals for cosmetics has been banned since 2013 (EC, 2009b). Within this regulatory area, *in vivo* data may only be used for the hazard assessment when already available. Although not explicitly banned, acute toxicity testing is discouraged for medicinal products, because the information gained from such studies is often of limited value for medicinal products and can be obtained from other toxicity studies (e.g. range finding studies for long-term experiments) (EMA, 2010). Compared to the cosmetics legislation, REACH, biocides, as well as food and feed regulations, have few testing requirements for toxicokinetic information (e.g. dermal and oral adsorption, distribution and persistence in the body, etc.). Further, REACH also lacks specific information requirements related to certain mechanistic toxicological endpoints such as immunotoxicity, although such concerns may be addressed by Substance Evaluation.

#### 2.4.3 Biotic systems and environment

Legislation for cosmetics and medical devices has no explicit information requirements for effects on biotic systems (i.e. ecotoxicity) and environmental fate and behaviour of substances (Table 1), as they refer to other legislation (REACH and medicinal products, respectively) to address these aspects. The environmental information requirements for REACH, biocides, and food and feed (including plant protection products) are clearly more comprehensive than those for medicinal products. Among those three areas (i.e. REACH, biocides and food and feed), there are only a few differences in ecotoxicity and environmental information requirements.

The overview in Table 1 also shows that most information requirements are shared by multiple or even all regulatory areas. Where differences exist, this can relate to differences in the intended applications or uses of chemicals regulated by the different pieces of legislation. In other cases, differences result from the fact that regulatory areas refer to other regulatory areas to cover the assessment of certain risks of a substance (e.g. for requirements related to the environmental and biotic systems, cosmetics and medical devices directives rely on REACH and medicinal products, respectively). It should be noted that many information requirements are conditional. In REACH, for example, the information requirements for chemicals depend on the annual production/import tonnage. All areas also use a tiered or context dependent approach for information requirements, i.e. the information required depends on the availability and waiving of toxicity studies or, for instance, the application or use of a chemical (e.g. if the inhalation route is not relevant, no information on this exposure route is required). Some studies and tests are more frequently conducted and/or are more broadly applicable across multiple areas than others. This needs to be considered in the prioritisation of future research needs and identification of the type of action that is needed.

## 3 Overview of completed and ongoing projects at OECD and further needs for actions

For each identified information requirement, RIVM and EU experts evaluated whether there is a need for adaptation or development of TGs/GDs to provide the required information for nanomaterials. This section gives a brief overview of the identified needs for further actions. An extended summary of the identification of needs for further actions for each information requirement is provided in the Supplementary information (Tables S3–S5).

Before highlighting further needs for action we first provide an overview of recently published OECD documents, followed by an overview of ongoing projects towards development or adaptation of OECD TGs or GDs. For all of the information requirements for which TGs/GDs have been adapted or are being addressed in ongoing projects we concluded that there is no (likely) or immediate need for further actions.

### 3.1 Recent (adaptations to) OECD TGs/GDs for nanomaterials

For 19 of the 136 information requirements that were identified, new or updated OECD TGs/GDs have already been published. Since 2017, OECD published several new and updated TGs/GDs for nanomaterials to identify and address some of the most urgent regulatory needs (as identified by the OECD WPMN in several workshops) (OECD, 2009; OECD, 2014a; OECD, 2014b; OECD, 2014c; OECD, 2016a; OECD, 2016b). Through these documents, some of the most urgent regulatory needs (from an EU perspective) for nanomaterial testing are currently addressed by legislation or related guidance. Documents include updates of TG 412 (OECD, 2017a), TG 413 (OECD, 2017b), and GD 39 (OECD, 2018) for inhalation toxicity, as inhalation exposure to nanomaterials is generally deemed to be of the highest human health concern.

For environmental fate and behaviour endpoints, a new TG on dispersion stability was published (TG 318) (Kozin and von der Kammer, 2017; OECD, 2017c), accompanied by further guidance (GD 318) (Ahtiainen, 2020; OECD, 2020). Testing leaching in soil columns (TG 312; OECD, 2004) is clarified for nanomaterials in GD 342 (OECD, 2021c). First steps towards a test guideline on nanomaterial removal from wastewater was captured in a study report (OECD, 2021b), although further work on the topic is needed, including (further) validation and/or modification of the test method.

For aquatic toxicity testing of nanomaterials the overarching GD 317 (OECD, 2021a) was published to provide adaptations for nanomaterials to enable applying a range of existing TGs for testing aquatic or sediment toxicity of nanomaterials, e.g. in daphnids (e.g. TG 202, TG211), algae (e.g. TG 201), fish (e.g. TG 203, TG229), or chironomids (e.g. TG 218, TG219). Additional OECD GDs were developed in OECD WPMN on physico-chemical properties (OECD, 2019a; OECD, 2019b) and exposure assessment (OECD, 2021d; OECD, 2021e; OECD, 2021f; OECD, 2021g). In April 2022, OECD approved a new TG for determination of the volume specific surface area of manufactured nanomaterials (OECD, 2022a) and a TG for nanomaterial particle size and particle size distribution of nanomaterials (OECD, 2022b).

### 3.2 Ongoing activities

In addition to these recent publications, new developments are still ongoing (Heunisch et al., 2022; OECD, 2022c). Some of these projects are supported by EU projects (e.g. Gov4Nano, 2019; NanoHarmony, 2020) or by national funding (Ahtiainen, 2020). These projects aim to adjust current test methods to nanomaterials or develop new approaches and/or perform interlaboratory comparisons. Among the activities included in the WNT workplan are TGs/GDs on physico-chemical properties (i.e. on dissolution, surface chemistry, surface hydrophobicity, and dustiness). These activities are expected to be completed in 2023, 2024, or 2025 (OECD, 2022c). For human health endpoints, projects are ongoing on genotoxicity (WNT Project 4.95), intestinal fate (WNT Project 4.158), and toxicokinetics (WNT Project 4.146). In addition, a study report on skin sensitisation testing needs for nanomaterials is in preparation (WNT Project 4.133). These projects are also expected to be finalised in the coming years. For environmental safety, ongoing activities include transformation (WNT Project 3.16), dissolution (WNT Project 3.10), heteroagglomeration (German Environmental Agency (UBA), 2022), and bioaccumulation (WNT Project 3.12), as well as further guidance on acute aquatic effect testing (in WPMN) (OECD, 2022c). An overarching project on the determination of concentrations of nanoparticles in biological samples (WNT Project 1.10) (OECD, 2022c) is also ongoing. A recent report provides further details on these ongoing OECD developments (Heunisch et al., 2022).

### 3.3 Needs for further action

Our analysis shows that for 42 of the 136 information requirements, there is no (likely) or immediate need for further action. A need for further action was identified for 62 information requirements. These were subsequently categorised into one of the following three groups.

1. Potential needs specific to nanomaterials and relevant to multiple regulatory areas (22 information requirements).
2. Information requirements for which the specific need for further work remains unclear and requires further investigation (29 information requirements).
3. Potential needs specific to nanomaterials but not broadly relevant, e.g. relevant to a specific requirement in only one regulatory area, or requirements not routinely used in risk assessment (11 information requirements)

We conducted this categorisation for prioritisation purposes. Table 2 provides a summary of the expert views on potential needs in the first group, i.e. the needs that are specific for nanomaterials and broadly relevant. Information requirements for which the specific need for further work remains unclear (i.e. the second group) are summarised in Table 3. These topics may require further investigation. Potential needs specific to nanomaterials but which are not broadly relevant (i.e. the third group) are not further discussed in detail in this document but can be found in the Supplementary information (Table S5). Extended summaries of the identified potential needs identified of each of the three groups are provided in the Supplementary Information (Tables S3–S5).

**Table 2:** Summary of EU and RIVM expert opinions on the potential needs that are specific for nanomaterials and relevant to multiple regulatory areas (note that the food and feed area include plant protection products). Information requirements (in bold) with similar needs are grouped in single rows and separated by “/”.

Information requirements	Summary of expert opinions on nano specific needs	Relevant OECD TGs/GDs^a^
**Physico-chemical properties**		

**Dispersion stability in relevant media**, required for: - REACH - Cosmetics - Food and feed - Biocides - Medicinal products - Medical devices	This endpoint is addressed in TG 318 and GD 318 for environmental media but further action on standardisation for biological media used in toxicology studies relevant for human health is needed, including *in vitro* studies.	TG 318
		GD 318
**Stability (physical and chemical)**, required for: - Cosmetics - Food and feed - Biocides - Medicinal products - Medical devices - Veterinary medicinal products	This endpoint need is relevant for all (eco)toxicity and *in vitro* studies during exposure.	–

**Health effects**		

**Reactivity** (catalytic activity, chemical reactivity, photocatalytic activity or radical formation potential), required for: - Cosmetics - Food and feed - Biocides - Medical devices - Veterinary medicinal products	Legislation is generally not very specific on methods to be used for these endpoints, although OECD TG 442C (in addition to several ISO documents,^b^ has been mentioned in this context. There is also a clear link with the oxidising/redox properties.	TG 442C
	Measurements of reactive oxygen species (ROS) need to be further optimised and potentially standardised (for both acellular and cellular assays), with consideration for potential assay interference of nanomaterials.	

**Cell toxicity (damage to cell/cell membrane, growth, metabolism)**, required for: - Cosmetics - Food and feed - Medical devices - Veterinary medicinal products	As *in vitro* assays are increasingly relevant for IATAs/ITSs^c^, there is a need for further guidance on cellular *in vitro* assays in general, addressing issues such as colorimetric interference, media depletion, dosing, sedimentation, exposure periods, target cell selection. Many of these issues are specifically relevant when testing (nano)particles.	–

**Inflammation induction (*in vitro*)**, required for: - Cosmetics - Food and feed - Medical devices	Induction of inflammation is considered as a central effect of solid (nano)particles, but there is no standardised method currently available. Work towards this goal is in progress in multiple EU (nano) projects.	

Mutagenicity: ***In vitro* cytogenicity study in mammalian cells or *in vitro* micronucleus study/In case of positive results *in vitro, in vivo* genotoxicity study (somatic and potentially germ cell**), required for: - All regulatory areas	Some OECD tests require adaptation for nanomaterials whereas applicability of other TGs for nanomaterials is uncertain. Focus is needed on whether particles are taken up and/or reach the cell/nucleus. There are on-going initiatives (e.g. hesiglobal.org/genetic-toxicology-gttc) to develop a protocol for genetic toxicity testing of nanomaterials.	TG 475
		TG 483
		TG 486
		TG 487
		TG 488
		TG 489
Acute toxicity (oral/inhalation/dermal route), required for: - REACH - Food and feed - Biocides - Medical devices - Veterinary medicinal products	To date, only TGs for subacute and subchronic toxicity have been adapted for nanomaterials. As inhalation is of the highest priority, there is a need to investigate and adapt the remaining protocols with regard to dosing, administration, toxicity criteria and 3R (replacement, reduction, refinement) compliance where GD 39 is not adequate. Acute oral and dermal testing of nanomaterials are currently of less relevance but may become more important in future.	TG 402
		TG 403
		TG 420
		TG 423
		TG 425
		TG 427
		TG 433
		TG 436
		GD 39
**Phototoxicity**, relevant for: - Cosmetics - Biocides - Medicinal products	Phototoxicity and photogenotoxicity need further action and TG update to accommodate to nanomaterials	TG 432

**Effects on biotic systems**		

Effects on, specifically, terrestrial organism: **Short-term toxicity to invertebrates/Effects on soil micro-organisms/Short-term toxicity to plants/Long-term toxicity testing on invertebrates/Long-term toxicity testing on plants**, required for: - REACH - Biocides - Food and feed - Medicinal products - Veterinary medicinal products	So far, activities have been focused on aquatic environment. Adaptations for soil testing of nanomaterials are needed, specifically related to dosing and determining actual doses. An overarching document similar to GD 317 may be required.	TG 207
		TG 208
		TG 216
		TG 220
		TG 222
		TG 226
		TG 227
		TG 228
		TG 232
		TG 317
Environmental fate and behaviour		
**Biotic degradation/Ready biodegradability/Simulation testing on ultimate degradation in surface water/Soil simulation testing/Biodegradation in manure/Sediment simulation testing**, required for: - REACH - Biocides - Food and feed - Medicinal products - Veterinary medicinal product	There is a need to adapt/develop TGs/GDs to provide information on the biotic degradation for nanomaterials (specifically relevant for nanomaterials with organic, organometallic components or coatings).	TG 301
		TG 302B
		TG 302C
		TG 307
		TG 308
		TG 309
		TG 310
		TG 311
		TG 320
**Biological water remediation: aerobic and anaerobic biodegradation; Sewage treatment works simulation test/Biodegradation in marine water**, required for: - Biocides - Food and feed - Medicinal products - Veterinary medicinal products	There is a need for research on the validity of the developed sewage treatment works method for nanomaterials (OECD, 2021b) (specifically relevant for nanomaterials with organic, organometallic components or coatings). Further, there is a need to introduce hetero-agglomeration into TG 318 and GD 318.	TG 303
		TG 209
		TG 306
		TG 318
		GD 318

a These OECD documents are specifically referenced in one or more of the documents used in identifying the regulatory requirements (see Supplementary Information Table S2) or identified by experts. Listing them here should not be interpreted as a need to adapt all of these documents for nanomaterials but rather as an overview of relevant OECD documents for which the needs for adaptation requires investigation. Current versions of these OECD documents are publicly available online: https://www.oecd.org/science/nanosafety/publications-series-safety-manufactured-nanomaterials.htmhttps://www.oecd.org/env/ehs/testing/oecdguidelinesforthetestingofchemicals.htm.

b Reactivity relevant ISO documents include ISO/TS 18827:2017, ISO/TS 19006:2016, ISO 20814:2019.

c IATAs, Integrated Approaches to Testing and Assessment; ITSs, Integrated Testing Strategies.

**Table 3:** Summary of EU and RIVM expert opinions on regulatory requirements for which the specific need for further work remains unclear and requires further investigation. Information requirements (in bold) with similar needs are grouped in single rows and separated by “/”.

Endpoint	Nano specific issue/need	Relevant OECD TGs/GDs^a^
**Physical-chemical properties**		

**Oxidising properties**, required for: - REACH - Cosmetics - Food and feed - Biocides	Test methods for oxidising properties are not applicable to nanomaterials, but their potential contribution to combustion of another material is relevant for the hazard assessment.	

**Relative density**, required for: - All regulatory areas	The current guideline may not be sufficient for nanomaterial inhalation studies, effective density may be more relevant than mass density.	TG 109

**Health effects**		

Skin/eye irritation/damage: **Skin/eye irritation/damage: Skin corrosion/irritation (*in vivo*)/Skin corrosion (*in vitro*)/Skin irritation (*in vitro*)/Serious eye damage/eye irritation (*in vivo*)/Serious eye damage/eye irritation (*in vitro*)**, required for: - All regulatory areas	Applicability for nanomaterials of available OECD TGs is uncertain/has not been investigated. Potential issues are likely related to dispersibility and dosing.	TG 404
		TG 405
		TG 430
		TG 431
		TG 437
		TG 438
		TG 439
		TG 435
		TG 460
		TG 491
		TG 492
		TG 494
		TG 496
**Skin sensitisation (*in vitro*/*in chemico*)/Skin sensitisation (*in vivo*)**, required for: - REACH - Cosmetics - Biocides - Medicinal products - Medical devices - Veterinary medicinal products	It is not clear to what extent nanospecific issues are anticipated. Applicability and adaptation needs for nanomaterials of existing *in vitro* skin sensitising tests is already under investigation. Further needs are not clear.	TG 442A
		TG 442B
		TG 442C
		TG 442D
		TG 442E
		TG 406
		TG 429
**Dermal absorption (*in vitro*)**, required for: - Cosmetics - Food and feed - Biocides - Medicinal products	It is not clear to what extent methods (existing or in development) are useful for nanomaterials. A systematic evaluation of appropriateness of such methods and guidance documents may be needed. Measurement of nanomaterials in biological tissues is a likely issue here (see also WNT Project 1.10).	TG 428

**Reproductive toxicity** (includes four information requirements, see Table S2 for details), required for: - All regulatory areas	Nanomaterials may pass through the placenta. Thus, information on the reproduction toxicity and on fertility/developmental effects is considered relevant. As for other TGs clear guidance on dispersion is important, as well as measurement of nanomaterials in biological tissues (see also WNT Project 1.10).	TG 414
		TG 415
		TG 416
		TG 421
		TG 422
		TG 443
**Endocrine disruption**, required for: - Cosmetics - Food and feed - Biocides	Potential endocrine disruption properties of nanomaterials may potentially be related to the particle properties or to properties of (released) chemical components of a nanomaterial (e.g. a coating). It is unclear which of the properties are most relevant.	TG 230
		TG 231
		TG 234
		TG 440
		TG 441
		TG 455
		TG 456
		TG 493
		TG 455
		TG 456
		TG 458
		TG 493
		GD 150
**Neurotoxicity**, required for: - Food and feed - Biocides - Medicinal products - Veterinary medicinal products - (REACH^b^)	It is not clear whether TGs are applicable to nanomaterials. There may be issues with dispersion stability and dosing.	TG 418
		TG 419
		TG 424
		TG 426
**Immunotoxicity**, required for: - Food and feed - Biocides - Medicinal products - Medical devices - Veterinary products - (REACH^b^)	Even for conventional chemicals guidance for immunotoxicity is not available. Adverse Outcome Pathway (AOP) key events related to interfering with the immune system are still being identified. Applicability of such AOPs for nanomaterials is unclear.	–

**Effects on biotic systems**		
**Activated sludge respiration inhibition testing**, required for: - REACH - Biocides - Medicinal products	There may be a need for adaptation or additional guidance for OECD TG 209.	TG 209
		TG 224
**Long-term toxicity testing on aquatic invertebrates (preferred species Daphnia)/Reproductive and development toxicity to an additional aquatic invertebrate species**, required for: - REACH - Biocides - Food and feed - Medicinal products - Veterinary medicinal products	OECD GD 317 already highlights issues with feeding, but more actions may be needed to sufficiently address issues with flow-through systems.	TG 211

**Long-term toxicity to sediment organisms**, required for: - REACH - Biocides - Food and feed - Human medicinal products - Veterinary medicinal products	OECD GD 317 may be sufficient, but there may be further needs for long-term toxicity testing to sediment organisms	TG 218
		TG 219
		TG 225
		TG 233
		TG 238
		TG 239
**Long-term or reproductive toxicity to birds/Acute oral toxicity to birds and mammals/Short-term dietary toxicity to birds/Toxic effects on livestock and pets/Food and feeding stuffs studies including for food-producing animals and their products (milk, eggs and honey)/Effects on other, non-target species (flora and fauna)**, required for: - REACH - Biocides - Food and feed	Guidance on human health endpoints may apply with regard to dosing.	GD 75
		TG 205
		TG 206
		TG 213
		TG 214
		TG 223
		TG 237
**Environmental fate and behaviour**		
Abiotic degradation: **Hydrolysis as a function of** pH, required for: - REACH - Biocides - Food and feed - Veterinary medicinal products	Unclear whether existing methods are applicable for nanomaterials.	TG 106
		TG 111
		TG 121
**Fate and behaviour in the environment**, required for: - REACH - Biocides - Food and feed - Medicinal products - Veterinary medicinal products	So far, most environmental OECD activities have been focussed on the aquatic environment, with soils receiving less attention. Yet, soils are a major sink for nanomaterials. There is a need to further examine fate and behaviour of nanomaterials in soils. It is unclear whether ongoing projects sufficiently address needs for nanomaterials and what additional action is needed (if any). In addition, there is a need to develop an alternative to the equilibrium partitioning method for nanomaterials to predict the distribution of nanomaterials in soils.	–

a These OECD documents are specifically referred to in one or more of the documents used in identifying the regulatory requirements (see Supplementary Information Table S2) or identified by experts. Current versions of these OECD documents are available online: https://www.oecd.org/science/nanosafety/publications-series-safety-manufactured-nanomaterials.htmhttps://www.oecd.org/env/ehs/testing/oecdguidelinesforthetestingofchemicals.htm.

b Note that effects on the development of nervous and immune systems can be measured in extended one-generation tests which is a (conditional) information requirement within REACH, but the decision to include these parameters is done on a case-by-case basis (ECHA, 2016).

Specific needs for further actions in Table 2, Table 3 are split into different sections that, as far as possible, align with the OECD sections for TGs. Some overarching themes for each section can be distinguished. For instance, regarding physico-chemical properties, development of guidance for assessment of the surface chemistry/reactivity of nanomaterials may require further efforts. For effects on biotic systems, potential issues are related to long-term testing (including interference of feed). Stability of the nanomaterials in relevant (environmental) media, (a)biotic degradation/transformation of nanomaterials with organic components, and interactions with natural (particulate) matter (adsorption/desorption, hetero-aggregation) are overarching issues that likely need further work for adequate environmental fate and behaviour assessment. Regarding human health endpoints, the main overarching issue appears to be related to sample preparation, impact of agglomeration, dispersion stability in biological media and the related dosing in toxicity testing. In addition, the dermal exposure route may need further action, depending on the potential for dermal barrier penetration. The latter may also play a role in skin sensitisation. Another, more overarching issue for both environmental and human health endpoints is related to the challenges in detecting and measuring (nano)particle concentrations in biological tissues. Although an ongoing project in OECD aims to provide guidance on this topic in the coming years, for some types of nanomaterials (e.g. carbon-based materials) further work is likely to be needed. Similar issues can be identified for determining concentrations and distinguishing (nano)particles in soil and sediment matrices.

## 4 Proposed prioritisation of further needs for actions

The current exercise identified potential needs (from a regulatory perspective) for development and adaptation of TGs and GDs to nanomaterials that are broadly relevant (see Table 2) and regulatory requirements for which the needs are uncertain and require further investigation (see Table 3). However, given the number of identified potential needs, further prioritisation of these identified needs is required. This calls for further discussion and scrutiny of existing knowledge among scientists, risk assessors and industry.

### 4.1 How can further activities be prioritised?

We believe that priority may be given to issues that are relevant for a range of regulatory requirements and/or a broader range of regulatory areas. Developing and/or adapting such broadly relevant TGs would also require a close collaboration between researchers and risk assessors from the different regulatory areas, as envisioned in the OECD TGP, in order to make any adaptations fit-for-purpose for all regulatory areas. When only a single regulatory area requires test adaptations for nanomaterials, a more focussed approach could be taken, e.g. by a targeted guidance development for the specific regulatory area, rather than the development of new OECD TGs or the adaptation of existing ones.

Apart from the number of regulatory areas, prioritisation may also be done based on the number of nanomaterials that require information on a specific regulatory requirement (e.g. when a whole range of carbon-based materials enter the market in large quantities, priority may need to be given to ensure these materials can be distinguished from biological tissues or soils). In the light of growing ethical requirements to reduce animal testing, priority should also be given to test methods/endpoints that allow waiving of vertebrate testing. The OECD MAD already helps to reduce the use of animals for regulatory purposes by minimising duplication of testing. Other approaches to reduce animal testing (i.e. New Approach Methodologies (NAMs) such as grouping and read-across) are needed and are currently being investigated and developed in various nanomaterial focussed EU projects, e.g. GRACIOUS (www.h2020gracious.eu), SUNSHINE (www.h2020sunshine.eu), or HARMLESS (www.harmless-project.eu), and in OECD activities, e.g. OECD QSAR Toolbox (www.oecd.org/chemicalsafety/risk-assessment/oecd-qsar-toolbox.htm), GD 211 (OECD, 2014e), GD 194 (OECD, 2014d). When it comes to the development or adaptation of TGs and GDs for nanomaterials, we believe that priority should be given to activities that are in support of such NAMs.

### 4.2 Proposed prioritisation for further action

Based on the above considerations, here we propose a prioritisation for future work. Table 2 shows that there are multiple information requirements that have similar needs for further action. For example, the importance of guidance on sample preparation, agglomeration and/or dispersion stability is mentioned for several physical-chemical properties (i.e. dispersion stability in relevant media, stability (physical and chemical)) and a human health information requirement (i.e. cell toxicity). For other information requirements the issue of sample preparation, agglomeration and/or dispersion stability was not specifically identified. However, it can be assumed that this issue also applies to other human health information requirements related to hazards identification (e.g. reactivity, mutagenicity, acute toxicity).

Further, the development of tests or guidance on degradation of organic nanomaterials (including all nanomaterials that contain carbon), and nanomaterials with organic components, appears to be a common need for further action among information requirements related to the environmental fate and behaviour and identifying (potential) degradation/transformation products that organisms might be exposed to in the environment. For organic nanomaterials, there are still major challenges with distinguishing them from complex (organic rich) matrices (e.g. soils and sediments) and assessing their degradation/transformation. Currently available methods are mostly developed for metal (oxide)/inorganic nanomaterials (e.g. single particle ICP-MS). Resolving this issue will be beneficial for all environmental toxicity testing, environmental fate modelling and potentially the assessment of toxicokinetics of organic materials in human matrices as well.

When considering needs for the development of NAMs, high-throughput (a)cellular measurement of the reactivity of nanomaterials appears to be vital. Such (a)cellular test systems can facilitate grouping and read-across approaches (Stone et al., 2020) and Safe-and-Sustainable-by-Design of future materials. Where NAMs (e.g. for testing key events in Adverse Outcome Pathways) are used in this context, such methods should enable clear regulatory decision making on the relevant regulatory endpoints.

Specifically, we therefore, propose to prioritise future work towards the following overarching issues.

1. Resolving issues around nanomaterial sample preparation, agglomeration, dispersion stability and dosing in toxicity testing, in particular for human health endpoints.
2. Further development of tests or guidance on degradation and transformation of organic nanomaterials or nanomaterials with carbon components to better assess environmental fate of this group of nanomaterials.
3. Further development of tests and guidance to measure (a)cellular reactivity of nanomaterials, which will be critical, e.g. for the development of NAMs and in high-throughput systems needed for assessing the ever increasing diversity of (newly) developed (advanced) nanomaterials

Focusing on these three overarching topics could help to resolve some of the most urgent outstanding scientific and regulatory issues for nanomaterials. The exact actions to take and identification of responsible stakeholders may be issue specific. For example, the issue of dispersion stability and dosing in toxicity testing for human health endpoints (i.e. the first of the suggested priority above) could be tackled by the development of an overarching GD based on the current knowledge (e.g. in line with (OECD, 2012) that is currently being updated). An alternative approach may be to develop a specific GD that outlines an approach similar to that of OECD GD 318 (OECD, 2020) described for environmental media. In contrast, degradation and transformation of organic nanomaterials (the second suggested priority above) and (a)cellular reactivity (the third suggested priority above) may require further research and additional data before specific tests or guidance can be developed.

## 5 Outlook and further considerations

The OECD Test Guidelines are fundamental to the any chemicals legislation globally, as they provide a set of regulatory recognised methods for testing chemicals that fall under the principle of Mutual Acceptance of Data in the Assessment of Chemicals^5^ provided that they are performed according to Good Laboratory Practice (GLP). Tests performed according to OECD test guidelines and GLP are recognised in countries adhering to MAD and this is an essential component for international harmonisation of approaches to chemical safety. Hence, an important task under the WPMN, and in close co-operation with the WNT, is to ensure that OECD Test Guidelines are applicable to nanomaterials. By identifying and prioritising needs for further work, we hope that the current analysis can contribute to the development and adaptation of OECD TGs for nanomaterials and, thereby, to contribute to safer use and production of nanomaterials.

### 5.1 Not all TGs need further actions

We would like to note that the identification of a need for further actions indicates that there is a need for harmonisation to improve comparability of results. It should not be interpreted as a rejection of the applicability of an existing OECD TG/GD for regulatory purposes, which would make it impossible to comply to certain regulatory requirements. In fact, many of the issues can be and are already being tackled by users of the current TGs, e.g. where they relate to the dosing of a test medium or test organism. For harmonised approaches, ensuring that results can be compared, and enabling the MAD, however, solutions to overcome the identified issues are best captured in the OECD documents themselves. Most of the needs for further action are pointing in such directions. Arguably, only in a few cases applicable methods for nanomaterials may be lacking.

Table 2, Table 3 identify relevant OECD TGs/GDs for each information requirement, but it should be emphasised that the aim of our current work was not necessarily to identify specific OECD documents to be worked on. Rather, scientific issues were identified that may be relevant for a certain information requirement or related test method. It should also be noted that such issues may not always relate to the whole range of nanomaterials. Issues may be restricted to certain types, e.g. only metals/metal oxides or only carbon-based nanomaterials. More scrutiny of scientific progress is needed to further prioritise follow-up actions in relation to both the applicability and readiness level of (new) test methods, and (the applicability for) specific types of nanomaterials (including e.g. new/advanced materials). This may also need to include an assessment of transformations of nanomaterials throughout the life cycle (including disposal and waste treatment) and specifically identifying the “worst-case” or representative test material among a variety of nanoforms.

### 5.2 Needs for further exchange and collaborative action

In this analysis, we included the opinions of experts working in the field of nanosafety and regulation of nanomaterials. Experts were invited to contribute to this work based on their expertise on nanosafety, their involvement in relevant international projects (e.g. within EU or OECD programmes) and their knowledge on different regulatory areas. Industry representatives were not included, and so, these perspectives may be missing in this analysis. Further, we acknowledge that more opinions (e.g. within industry but also within academia and regulators) may be present in the nanosafety community. Therefore, we encourage further scrutiny and broader discussion of the proposed prioritisation, in particular in forums or workshops where different opinions are likely to meet, e.g. in (workshops organised by) OECD WPMN, or the (European) risk assessment arena.

The European REFINE (Regulatory Science Framework for Nano(bio)material-based Medical Products and Devices) project recently published the results of an exercise similar to that presented here. In the study, the authors aim to identify methodological gaps associated with the preclinical characterisation for nanotechnology-based medicinal products and medical devices (Halamoda-Kenzaoui et al., 2019, 2021). For this reason, and because OECD TGs are not often used in pre-clinical risk assessments, we did not prioritise issues that are identified for the medicinal sector alone. Yet, we do acknowledge that certain issues that we identified for other regulatory areas may also be relevant for the medical area. We encourage that actions on such issues are picked up in close collaborations between the different areas. Such collaborations may also be relevant for issues that may not be directly relevant for the medical area, but for which (partial) solutions have been found in that medical area.

### 5.3 ‘Advanced materials’ should also be considered in further actions

The focus of this exercise was more on the conventional nanomaterials (i.e. as solid particles according to the 2022 recommendation (EC, 2022)). However, in the last two decades, progress in nanotechnology has resulted in the development of many nanomaterials with new functionalities and increasing complexity. Where previously relatively simple passive and active nanostructures have been developed, more complex and smart objects now emerge that combine different materials/chemistries into one structure. These complex products often incorporate materials into hybrid systems, e.g. bio-nano systems (Roco, 2018). Currently, such complex ‘advanced (nano)materials’ are gaining more attention in the scientific literature (Mech et al., 2022; Oomen et al., 2022; Schwirn et al., 2021). Yet, the chemical legislation and risk assessment, developed to assess individual substances, still generally focus on the more simple nanostructures, although current developments in the European regulatory arena (e.g. Green Deal (EC, 2019) and Chemical Strategy on Sustainability (EC, 2020)) may change the focus. Nevertheless, and irrespective of whether there is a clear regulatory need or not, we strongly recommend to closely monitor the development of these complex ‘advanced materials’ and encourage to include them in the applicability domain of any test guideline to be adapted or newly developed wherever possible. This will allow ‘future proofing’ of documents and minimises the need for further updates.

### 5.4 Further development of FAIR data(bases) is crucial

The aim of this paper was to identify the specific needs for further action (e.g. the development or adaptation of TGs/GDs) for specific information requirements to accommodate nano-specific issues. Ultimately, work towards the identified priorities could contribute to better nanosafety. However, safe production and application of nanomaterials relies not only on fit-for-purpose methods but also on the availability of data. To date, the nanosafety community has not been able to fully exploit the data that have been produced over the last decade. This is partly due to the lack of data management tools as well as the complexity of nanosafety data (Jeliazkova et al., 2021). Recently, multiple collaborative projects (e.g., eNanoMapper (Jeliazkova et al., 2015), NanoInformaTIX^6^) have built platforms that aim to make nanomaterial data accessible following the FAIR data guiding principles (Wilkinson et al., 2016). In the light of improving the safety and risk assessment of nanomaterials, there is, in addition to the need to focus on developing and adapting TGs/GDs, a strong need to further the development of FAIR data(bases) for nanomaterials.

### 5.5 Towards ‘one substance – one assessment’ in the EU

The current assessment also highlights that regulatory requirements among the seven considered EU regulatory areas are very similar. Differences exists, yet many of these relate to the differences in intended application and use of the chemical or product regulated within a particular legislation. For example, the number of information requirements for cosmetics and medicinal products are expanded for skin sensitisation and skin irritation compared to other regulatory areas which is related to that dermal application for these products is a major route of exposure in humans. The large overlap in information requirements among regulatory areas clearly points to the benefits of closer collaboration and harmonisation among the different regulatory areas, which is also the aim of the “one substance, one assessment” approach in Europe (EC, 2020). To anticipate the further integration of EU chemicals legislation, there is a need for closer collaboration between risk assessors, regulators and scientists of different areas not only in the use of data but also in the development and adaptation of TGs/GDs. This would ensure inclusion of knowledge from the different areas in the development of test methods and ensures that test methods will be fit-for-purpose for all of the different regulatory areas.

### 5.6 Integration of risk governance with innovation policy

Further, if legislation is adapted to specific requirements of (a group of) chemicals (e.g. nanomaterials), a precondition for the successful implementation of such legislation is the availability of suitable guidance and test guidelines. Uncertainty about regulatory validity could hamper the exploitation of the full economic potential and, thereby, limit the potential of nanotechnology to deliver on solutions for societal and environmental challenges such as those identified in the European Green Deal (EC, 2019). Therefore, we also call for a structural process of identification of information needs and knowledge generation, preferably as part of risk governance and closely connected to technological innovation policy. Such a structural process appears essential to enable a quick response to new innovations and any potential issues that may arise from those innovations, either in (regulatory) risk assessment or from the test methods that provide the scientific basis for such assessment. The case of nanomaterials has shown that without such a structure chances are high that risk governance is lagging far behind developments of new innovations and technologies, which ultimately hampers such innovation.

## 6 Conclusion

This work brought together the scientific opinion of a group of international nanosafety and chemicals regulatory area-specific experts. By comparing the information requirements of the different regulatory areas in the EU, a group of 22 experts identified common challenges in compliance to chemical legislation and guidance. We propose to prioritise work towards three topics (see above) that are relevant for multiple information requirements and/or across multiple regulatory areas. Work towards these topics could help to resolve some of the most urgent outstanding scientific and regulatory issues with regard to nanomaterials. As such, this paper may guide (further) actions towards improved safety and risk assessment of (advanced) nanomaterials and help prioritise these actions and identify the respective stakeholders (e.g. identify from which specific regulatory areas experts are to be involved in any future action). Although this analysis focused on EU legislation, the findings, linked as they are to international testing methods (e.g. OECD Test Guidelines), are anticipated to be relevant to regulatory regimes in other national and international contexts as well.

## Disclaimer

The content expressed in this paper is solely the opinion of the authors and does not necessarily reflect the opinion of their institutions.

## CRediT author statement

Conceptualization: EB; Requirements collection: JK, MV, EB ES; Investigation (i.e. identification of needs, feedback on initial overview): all authors; Formal analysis (i.e. synthesis of collected input): ES, EB; Writing - Original Draft: ES, EB, Writing – Review and Editing: all authors.

## Funding body information

This work was funded by 10.13039/501100007192the RIVM Risks of Nanotechnology Knowledge and Information Centre (KIR-nano) which is funded by 10.13039/501100002999the Netherlands Ministries Infrastructure and Water Management (I&W), Health, Welfare and Sport (VWS) and Social Affairs and Employment (SZW). Additional funding was received from the 10.13039/501100000903EU project NanoHarmony, which has received funding from the European Union's Horizon 2020 research and innovation programme under grant agreement No 885931.

## Declaration of competing interest

The authors declare that they have no known competing financial interests or personal relationships that could have appeared to influence the work reported in this paper.

## Data Availability

No data was used for the research described in the article.

## Footnotes

2 https://www.nanosafetycluster.eu/international-cooperation/the-malta-initiative.

3 https://echa.europa.eu/regulations/nanomaterials/nanomaterials-expert-group.

4 https://echa.europa.eu/documents/10162/2580982/nmeg-9_minutes_final_en.pdfhttps://echa.europa.eu/documents/10162/1785753/nmeg-10_minutes_open_session_en.pdf.

5 See http://www.oecd.org/env/ehs/mutualacceptanceofdatamad.htm.

6 www.nanoinformatix.eu.
