# Supplementary material for: Towards harmonisation of testing of nanomaterials for EU regulatory requirements on chemical safety – A proposal for further actions
Source: Regul Toxicol Pharmacol. 2023 Mar;139:105360. doi: 10.1016/j.yrtph.2023.105360 (PMC10060946; doi:10.1016/j.yrtph.2023.105360)
Supplement: Multimedia component 1 [file mmc1.docx]

Towards Harmonisation of Testing of Nanomaterials for EU Regulatory Requirements on Chemical Safety – A Proposal for Further Actions –Supplementary Information

# **Authors:**

Eric A.J. Bleeker^1*^, Elmer Swart^1*^, Hedwig Braakhuis^1^, María Luisa Fernández-Cruz^2^, Steffi Friedrichs^3^, Ilse Gosens^1^, Frank Herzberg^4^, Keld Alstrup Jensen^5^, Frank von der Kammer^6^, Jolinde A.B. Kettelarij^1^, Jose María Navas^2^, Kirsten Rasmussen^7^, Kathrin Schwirn^8^, Maaike Visser^1^

^1^ National Institute for Public Health and the Environment (RIVM), P.O. box 1, 3720 BA Bilthoven, The Netherlands

^2^ Instituto Nacional de Investigación y Tecnología Agraria y Alimentaria (INIA), CSIC. Ctra. De la Coruña Km 7,5 28040 Madrid, Spain

^3^ AcumenIST SRL, Rue Fétis 19, 1040 Etterbeek, Belgium

^4^ German Federal Institute for Risk Assessment (BfR), Max-Dohrn-Str. 8-10, 10589 Berlin, Germany

^5^ The National Research Centre for the Working Environment (NRCWE), 105 Lersø Parkallé, DK-2100 Copenhagen

^6^ University of Vienna, Centre for Microbiology and Environmental Systems Science, Department of Environmental Geosciences, Josef-Holaubek-Platz 2, 1090 Vienna, Austria

^7^ European Commission, Joint Research Centre (JRC), Ispra, Italy

^8^ German Environment Agency (UBA), Woerlitzer Platz 1, 06844 Dessau-Rosslau; Germany

**Table S1**: Overview of EU regulatory documents considered for the identification of regulatory requirements for nanomaterials.

| Regulatory area | Considered documents |
| --- | --- |
| Industrial chemicals | European regulation on chemicals (REACH) (Regulation (EC) 1907/2006) (EC, 2006) |
|  | Amendments of the REACH regulation to address nanoforms (Regulation (EU) 2018/1881) (EU, 2018) |
|  | ECHA guidance (https://echa.europa.eu/guidance-documents/guidance-on-reach) |
| Cosmetics | European regulation on cosmetic products (Regulation (EC) 1223/2009) (EC, 2009) |
|  | SCCS notes of guidance for the testing of cosmetic ingredients and their safety evaluation (SCCS, 2018) |
|  | SCCS guidance on the safety assessment of nanomaterials in cosmetics (SCCS, 2019) |
| Food and feed | European regulation on food information to consumers (Regulation (EU) 1169/2011) (EU, 2011) |
|  | European regulation on data requirements for active substances in plant production products (Regulation (EU) 283/2013) (EU, 2013) |
|  | European regulation on novel foods (Regulation (EC) 2015/2283) (EC, 2015) |
|  | EFSA guidance for submission for food additive evaluations (EFSA Panel on Food Additives Nutrient Sources added to Food, 2012) |
|  | EFSA guidance to safety of feed additives for the environment (EFSA FEEDAP Panel et al., 2019) |
|  | EFSA guidance on risk assessment of nanomaterials to be applied in the food and feed chain: human and animal health 2021 (EFSA Scientific Committee et al., 2021) |
| Biocides | European regulation on biocidal products (Regulation (EU) 528/2012) (EU, 2012) |
|  | ECHA guidance (https://echa.europa.eu/guidance-documents/guidance-on-biocides-legislation) |
| Medicinal products | European directive on directive medicinal products for human use (Directive (EC) 2001/83) (EC, 2001) |
|  | EMA note for guidance on test procedures for new drugs (chemical substances) (EMEA, 2000) |
|  | EMA note for guidance on testing carcinogenic potential (EMEA, 2002) |
|  | EMA guideline on the non-clinical investigation of the dependence potential of medicinal products (EMEA, 2006b) |
|  | EMA note for guidance on immunotoxicity studies for human pharmaceuticals (EMEA, 2006c) |
|  | EMA guideline on environmental risk assessment of medicinal products for human use (EMEA, 2006a) |
|  | EMA note for guidance on genotoxicity testing and data interpretation for pharmaceuticals intended for human use (EMEA, 2008c) |
|  | EMA guideline on risk assessment of medicinal products on human reproduction and lactation (EMEA, 2008a) |
|  | EMA guideline on the need for non-clinical testing in juvenile animals on human pharmaceuticals for paediatric indications (EMEA, 2008b) |
|  | EMA guideline on repeated dose toxicity (EMA, 2010) |
|  | MHLW/EMA reflection paper on the development of block copolymer micelle medicinal products (EMA, 2013a) |
|  | EMA reflection paper on the data requirements for intravenous liposomal products (EMA, 2013b) |
|  | EMA guidance on photosafety evaluation of pharmaceuticals (EMA, 2015b) |
|  | EMA guideline on non-clinical local tolerance testing of medicinal products (EMA, 2015a) |
|  | EMA reflection paper on the data requirements for intravenous iron-based nano-colloidal products (EMA, 2015c) |
|  | EMA guideline on the chemistry of active substances (EMA, 2016) |
|  | EMA guideline on the chemical and pharmaceutical quality in clinical trials (EMA, 2017) |
|  | EMA guideline on the environmental risk assessment of medicinal products for human use (EMA, 2018) |
|  | EMA guideline on reproductive toxicology for human pharmaceuticals (EMA, 2020) |
| Medical devices | European regulation on medical devices (Regulation (EU) 2017/745) (EU, 2017) |
|  | SCENIHR guidance on nanomaterials in medical devices (SCENIHR, 2015) |
|  | ISO guidance on immunotoxicology testing of medical devices: (ISO, 2006) |
|  | ISO guidance on tests for in vitro cytotoxicity of medical devices (ISO, 2009) |
|  | ISO guidance on tests for genotoxicity, carcinogenicity and reproductive toxicity of medical devices (ISO, 2014) |
|  | ISO guidance on tests for systemic toxicity of medical devices (ISO, 2017a) |
|  | ISO guidance on biological evaluation of nanomaterials in medical devices (ISO, 2017b) |
|  | ISO guidance on tests for skin sensitization of medical devices (ISO, 2021a) |
|  | ISO guidance on tests for irritation of medical devices (ISO, 2021b) |
| Veterinary medicinal products | European regulation on veterinary medicinal products (Regulation (EU) 2019/6) (EU, 2019) |
|  | Amendment of Annex II of the European regulation on veterinary medicinal products (Delegated Regulation (EU) 2021/805) (EU, 2021) |
|  | EMA guidance on reproduction testing of residues of veterinary drugs in human food (EMEA, 2004a) |
|  | EMA guidance on repeated-dose 90 day toxicity testing of residues of veterinary drugs in human food (EMEA, 2004b) |
|  | EMA guidance on repeated-dose chronic toxicity testing of residues of veterinary drugs in human food (EMEA, 2004c) |
|  | EMA guidance on environmental impact assessment for veterinary medicinal products (EMEA, 2004d) |
|  | EMA guidance on human carcinogenicity testing of veterinary drugs (EMEA, 2005) |
|  | EMA guidance on a testing approach for residues of veterinary drugs in human food (EMEA, 2009) |
|  | EMA guidance on determining fate of veterinary medicinal products in manure (EMA, 2011) |
|  | EMA guidance on genotoxicity testing of residues of veterinary drugs in human food (EMA, 2014) |
|  | EMA advice on implementing the European regulation on veterinary medicinal products (EMA, 2019) |

ECHA: European Chemicals Agency; SCCS: Scientific Committee on Consumer Safety; EFSA: European Food Safety Authority; EMA: European Medical Agency; MHLW: Japanese Ministry of Health, Labour and Welfare; SCENIHR: Scientific Committee on Scientific Committee on Emerging and Newly Identified Health Risks; ISO: International Organization for Standardization.

**Table S2**: Overview of regulatory requirements for nanomaterials per EU regulatory area and expert assessment on potential need for further action. Tick marks indicate that the respective information requirement is included in a particular regulatory area. Published OECD documents are referenced and going actions in OECD and are further detailed in the section ‘Overview of the completed and ongoing projects in OECD and further needs for actions’ in the main document. Although information requirements are generally very similar, exact descriptions of information requirements often differ between the regulatory frameworks. Terminology used here is often based on the terminology in REACH. Note that requirements for REACH in this overview are limited to those mentioned in the Annexes VI-X in the REACH regulation (EC, 2006; EU, 2018) and that the food and feed regulatory area as depicted in this table includes several regulations along the food and feed chain, including PPPs and novel foods, see also the Supplementary information for a list of considered documents (Table S1).

| Information requirement | EU regulatory area | | | | | | |  |
| --- | --- | --- | --- | --- | --- | --- | --- | --- |
|  | REACH | Cosmetics | Food and feed | Biocides | Medicinal products for human use | Medical devices | Veterinary medicinal products | Expert assessment on needs |
| Physico-chemical properties – Chemical name or identifier and molecular structural properties | | | | | | | | |
| Name(s) in the IUPAC nomenclature or other international chemical name(s) | ✓ | ✓ | ✓ | ✓ | ✓ | ✓ | ✓ | No likely need for action |
| Other names (usual name, trade name, abbreviation) | ✓ | ✓ | ✓ | ✓ | ✓ | ✓ | ✓ | No need for action |
| EINECS or ELINCs number (if available and appropriate) | ✓ | ✓ | ✓ | ✓ | ✓ | ✓ | ✓ | No need for action |
| CAS name and CAS number (if available) | ✓ | ✓ | ✓ | ✓ | ✓ | ✓ | ✓ | No need for action |
| Other identity code (if available) | ✓ | ✓ | ✓ | ✓ | ✓ | ✓ | ✓ | No need for action |
| Information related to molecular and structural formula of each substance | ✓ | ✓ | ✓ | ✓ | ✓ | ✓ | ✓ | No need for action |
| Molecular and structural formula (including SMILES notation, if available) | ✓ | ✓ | ✓ | ✓ | ✓ | ✓ | ✓ | No need for action |
| Information on optical activity and typical ratio of (stereo) isomers (if applicable and appropriate) | ✓ | ✓ | ✓ | ✓ | ✓ | ✓ | ✓ | No need for action |
| Molecular weight or molecular weight range | ✓ | ✓ | ✓ | ✓ | ✓ | ✓ | ✓ | No need for action |
| Physico-chemical properties – Composition and (im)purities | | | | | | | | |
| Degree of purity (%) | ✓ | ✓ | ✓ | ✓ | ✓ | ✓ | ✓ | No need for action |
| Nature of impurities, including isomers and by-products | ✓ | ✓ | ✓ | ✓ | ✓ | ✓ | ✓ | No need for action |
| Percentage of (significant) main impurities | ✓ | ✓ | ✓ | ✓ | ✓ | ✓ | ✓ | No need for action |
| Nature and order of magnitude (… ppm, … %) of any additives (e.g. stabilising agents or inhibitors) | ✓ | ✓ | ✓ | ✓ | ✓ | ✓ | ✓ | No need for action |
| Spectral data (e.g. ultra-violet, infra-red, nuclear magnetic resonance or mass spectrum) | ✓ | ✓ | ✓ | ✓ | ✓ | ✓ | ✓ | No need for action |
| High-pressure liquid chromatogram, gas chromatogram | ✓ | ✓ | ✓ | ✓ | ✓ | ✓ | ✓ | No need for action |
| Description of the analytical methods or the appropriate bibliographical references for the identification of the substance and, where appropriate, for the identification of impurities and additives. This information shall be sufficient to allow the methods to be reproduced | ✓ | ✓ | ✓ | ✓ | ✓ | ✓ | ✓ | No need for action |
| Physico-chemical properties – Physical descriptors of nanoform (e.g. size, shape, surface) and production methods | | | | | | | | |
| The information in points 2.4.2 – 2.4.5 shall be clearly assigned to the different nanoforms or sets of similar nanoforms identified in point 2.4.1 | ✓ |  |  |  |  |  |  | No need for action |
| Names or other identifiers of the nanoforms (or sets of similar nanoforms) of the substance | ✓ | ✓ | ✓ | ✓ |  | ✓ |  | No need for action |
| Number based particle size distribution with indication of the number fraction of constituent particles in the size range within 1 nm – 100 nm | ✓ | ✓ | ✓ | ✓ | ✓ | ✓ | ✓ | TG 125 published (OECD, 2022b) |
| Description of surface functionalisation or treatment and identification of each agent including IUPAC name and CAS or EC number | ✓ | ✓ | ✓ |  | ✓ | ✓ |  | Actions ongoing |
| Shape, aspect ratio and other morphological characterisation: crystallinity, information on assembly structure including e.g. shell like structures or hollow structures, if appropriate | ✓ | ✓ | ✓ | ✓ | ✓ | ✓ | ✓ | Actions ongoing |
| Surface area (specific surface area by volume, specific surface area by mass or both) | ✓ | ✓ | ✓ |  | ✓ | ✓ |  | TG 124 published (OECD, 2022a) |
| Description of the analytical methods or the appropriate bibliographical references for the information elements in this sub-section. This information shall be sufficient to allow the methods to be reproduced. | ✓ | ✓ | ✓ | ✓ | ✓ | ✓ |  | No need for action |
| Method of manufacture (syntheses pathway) of active substance including information on starting materials and solvents including suppliers, specifications and commercial availability |  | ✓ | ✓ | ✓ | ✓ | ✓ | ✓ | No need for action |
| Analytical profile of representative batches (g/kg active substance) including information on content of the impurities |  | ✓ | ✓ | ✓ | ✓ | ✓ | ✓ | No need for action |
| The origin of the natural active substance or the precursor(s) of the active substance, e.g. an extract of a flower |  |  | ✓ | ✓ | ✓ | ✓ | ✓ | No need for action |
| Granulometry | ✓ | ✓ | ✓ | ✓ | ✓ | ✓ | ✓ | TG 125 published (OECD, 2022b) |
| Dustiness | ✓ | ✓ | ✓ | ✓ |  | ✓ |  | Actions ongoing |
| Porosity (if it relates to a function) |  | ✓ | ✓ |  |  | ✓ |  | Actions ongoing |
| Physico-chemical properties – Basic physical and chemical properties (e.g. melting/freezing/boiling point, vapour pressure, pH, *K*_OW,_ dissolution) | | | | | | | | |
| State of the substance at 20 °C and 101,3 kPa | ✓ | ✓ | ✓ | ✓ | ✓ | ✓ | ✓ | No need for action |
| Colour |  | ✓ | ✓ | ✓ | ✓ |  | ✓ | No need for action |
| Odour |  | ✓ |  | ✓ |  |  | ✓ | No need for action |
| pH |  | ✓ | ✓ | ✓ | ✓ | ✓ | ✓ | No need for action |
| Melting/freezing point | ✓ | ✓ | ✓ | ✓ | ✓ |  | ✓ | No need for action |
| Boiling point | ✓ | ✓ | ✓ | ✓ | ✓ |  | ✓ | No need for action |
| Relative density | ✓ | ✓ | ✓ | ✓ |  | ✓ | ✓ | Potential need for action |
| Vapour pressure | ✓ | ✓ | ✓ | ✓ | ✓ |  | ✓ | No likely need for action |
| Surface tension | ✓ |  |  | ✓ |  |  |  | No likely need for action |
| Partition coefficient n-octanol/water | ✓ | ✓ | ✓ | ✓ | ✓ | ✓ | ✓ | TG 318 (OECD, 2017) and GD 318 (OECD, 2017) published and ongoing actions |
| Viscosity | ✓ | ✓ | ✓ | ✓ | ✓ | ✓ | ✓ | No need for action |
| UV absorption |  | ✓ |  | ✓ |  |  |  | No likely need for action |
| Water solubility | ✓ | ✓ | ✓ | ✓ | ✓ | ✓ | ✓ | GD 318 (OECD, 2017) published and ongoing actions |
| Stability in organic solvents and identity of relevant degradation products | ✓ | ✓ |  | ✓ | ✓ | ✓ | ✓ | Potential need for action |
| Dissociation constant | ✓ | ✓ |  | ✓ | ✓ |  | ✓ | Potential need for action |
| In-use stability studies at clinically relevant concentrations and under relevant storage conditions |  | ✓ |  | ✓ | ✓ | ✓ | ✓ | Potential need for action |
| **Physico-chemical properties – Stability in relevant media** | | | | | | | | |
| Dispersion stability in relevant media | ✓ | ✓ | ✓ | ✓ | ✓ | ✓ |  | Potential need for action |
| Stability, both physical and chemical |  | ✓ | ✓ | ✓ | ✓ | ✓ | ✓ | Potential need for action |
| Stability in lysosomal fluid |  | ✓ | ✓ |  |  |  |  | Ongoing |
| Physico-chemical properties – Flammability and explosive properties | | | | | | | | |
| Flash point | ✓ | ✓ |  | ✓ |  |  |  | No need for action |
| Flammability | ✓ |  |  | ✓ |  | ✓ |  | No need for action |
| Explosive properties | ✓ |  |  | ✓ |  | ✓ |  | No need for action |
| Self-ignition temperature | ✓ | ✓ |  | ✓ |  |  |  | No need for action |
| Oxidising properties | ✓ | ✓ | ✓ | ✓ |  |  |  | Potential need for action |
| Physico-chemical properties – Interaction with drugs and other active ingredients | | | | | | | | |
| Drug loading efficiency |  |  |  |  | ✓ |  |  | Potential need for action |
| Assay and distribution of any active ingredient associated with the nanomaterial and free in solution (e.g., surface-bound or liposome encapsulated versus free active ingredient) |  |  |  |  | ✓ | ✓ |  | Potential need for action |
| *In vitro* drug substance /siRNA release rate in physiologically/clinically relevant media |  |  |  |  | ✓ | ✓ | ✓ | Potential need for action |
| Health effects – Sensitization, irritation and inflammation | | | | | | | | |
| Inflammation induction (*in vitro*) |  | ✓ | ✓ |  |  |  |  | Potential need for action |
| Skin corrosion/irritation (*in vivo*) | ✓ | ✓ |  | ✓ | ✓ | ✓ | ✓ | Potential need for action |
| Skin corrosion (*in vitro*) | ✓ | ✓ |  |  | ✓ |  |  | Potential need for action |
| Skin irritation (*in vitro*) | ✓ | ✓ | ✓ |  | ✓ | ✓ |  | Potential need for action |
| Serious eye damage/eye irritation (*in vivo*) | ✓ | ✓ | ✓ | ✓ | ✓ | ✓ | ✓ | Potential need for action |
| Serious eye damage/eye irritation (*in vitro*) | ✓ | ✓ |  |  | ✓ | ✓ |  | Potential need for action |
| Skin sensitisation (*in vitro*/*in chemico*) | ✓ | ✓ |  |  | ✓ | ✓ |  | Potential need for action |
| Skin sensitisation (*in vivo*) | ✓ |  |  |  | ✓ | ✓ |  | Potential need for action |
| Respiratory sensitisation |  |  |  | ✓ |  |  | ✓ | No likely need for action |
| Phototoxicity |  | ✓ |  | ✓ | ✓ |  |  | Potential need for action |
| Health effects – Cytotoxicity and reactivity | | | | | | | | |
| Cell toxicity (damage to cell / cell membrane, growth, metabolism) |  | ✓ | ✓ |  |  | ✓ | ✓ | Potential need for action |
| Gastrointestinal barrier integrity impairment *in vitro* |  |  | ✓ |  |  |  |  | Ongoing |
| Reactivity (chemical reactivity, photocatalytic activity and radical formation potential) |  | ✓ | ✓ | ✓ |  | ✓ | ✓ | Potential need for action |
| Health effects – Genotoxicity, mutagenicity, carcinogenicity | | | | | | | | |
| *In vitro* gene mutation study in bacteria | ✓ | ✓ | ✓ | ✓ | ✓ | ✓ | ✓ | No likely need for action |
| *In vitro* cytogenicity study in mammalian cells or *in vitro* micronucleus study | ✓ | ✓ | ✓ | ✓ | ✓ | ✓ | ✓ | Potential need for action |
| *In vitro* gene mutation study in mammalian cells, if a negative result in Annex VII, Section 8.4.1. and Annex VIII, Section 8.4.2 | ✓ | ✓ | ✓ | ✓ | ✓ | ✓ | ✓ | Ongoing |
| In case of positive results *in vitro*, in vivo genotoxicity study (somatic and potentially germ cell) | ✓ |  | ✓ | ✓ | ✓ | ✓ | ✓ | Potential need for action |
| Carcinogenicity study | ✓ | ✓ | ✓ | ✓ | ✓ | ✓ | ✓ | No likely need for action |
| Health effects – Acute/short term toxicity | | | | | | | | |
| By oral route | ✓ |  | ✓ | ✓ |  | ✓ | ✓ | Ongoing |
| By inhalation | ✓ |  | ✓ | ✓ |  | ✓ | ✓ | GD 39 (OECD, 2018c) published |
| By dermal route | ✓ |  | ✓ | ✓ |  | ✓ | ✓ | Potential need for further action |
| Short-term repeated dose toxicity study (28 days), one species, male and female, most appropriate route of administration, having regard to the likely route of human exposure | ✓ | ✓ | ✓ | ✓ | ✓ | ✓ |  | TG 412 published (OECD, 2018a) |
| Health effects – Long term toxicity (e.g. reproductive toxicity) | | | | | | | | |
| Sub-chronic toxicity study (90-day), one species, rodent, male and female, most appropriate route of administration, having regard to the likely route of human exposure (specific additional endpoints include developmental neurotoxicity, immunotoxicity and endocrine disruption). | ✓ | ✓ | ✓ | ✓ |  | ✓ | ✓ | TG 413 published (OECD, 2018b) |
| Long term study (> 12 months); if applicable | ✓ |  | ✓ | ✓ | ✓ | ✓ |  | No likely need for action |
| Screening for reproductive/developmental toxicity, one species (OECD 421 or 422), if there is no evidence from available information on structurally related substances, from (Q)SAR estimates or from *in vitro* methods that the substance may be a developmental toxicant | ✓ | ✓ | ✓ |  |  | ✓ |  | Potential need for further action |
| Pre-natal developmental toxicity study, one species, most appropriate route of administration, having regard to the likely route of human exposure | ✓ | ✓ | ✓ | ✓ | ✓ | ✓ | ✓ | Potential need for further action |
| Extended one-generation reproductive toxicity, including the following endpoints:   - reproductive endpoints - developmental (prenatal and postnatal) endpoints - specific endpoints (developmental neurotoxicity, immunotoxicity and endocrine disruption) | ✓ |  | ✓ |  | ✓ | ✓ |  | Potential need for further action |
| Two generation reproductive toxicity study (if the available repeated dose toxicity studies (e.g. 28-day or 90-day studies) indicate adverse effects on reproductive organs or tissues or reveal other concerns in relation with reproductive toxicity.) | ✓ | ✓ | ✓ | ✓ | ✓ | ✓ | ✓ | Potential need for further action |
| Health effects – Uptake/kinetics | | | | | | | | |
| Dermal absorption (*in vitro*) |  | ✓ | ✓ | ✓ |  |  |  | Potential need for further action |
| Assessment of the toxicokinetic behaviour of the substance to the extent that can be derived from the relevant available information | ✓ | ✓ |  |  | ✓ | ✓ | ✓ | Ongoing |
| Oral absorption (*in vitro*) |  | ✓ |  | ✓ |  |  |  | Ongoing |
| Pharmacokinetic parameters |  |  |  |  | ✓ |  | ✓ | Potential need for further action |
| Health effects – Other requirements (incl. endocrine disruption, neurotoxicity, immunotoxicity, microbiome interactions etc.) | | | | | | | | |
| Endocrine disruption |  | ✓ | ✓ | ✓ |  |  |  | Potential need for further action |
| Neurotoxicity | ✓ |  | ✓ | ✓ | ✓ |  | ✓ | Potential need for further action |
| Immunotoxicity | ✓ |  | ✓ | ✓ | ✓ | ✓ | ✓ | Potential need for further action |
| Interaction with intestinal microbiome |  |  | ✓ |  |  |  | ✓ | No likely need for action |
| Use of data in humans |  | ✓ | ✓ | ✓ |  |  | ✓ | No likely need for action |
| Bioburden control |  |  |  |  |  |  |  | Potential need for further action |
| Pharmacodynamical parameters |  |  |  |  | ✓ |  | ✓ | Potential need for further action |
| Effects on biotic systems – Invertebrate toxicity | | | | | | | | |
| Short-term toxicity testing on invertebrates (preferred species Daphnia) | ✓ |  | ✓ | ✓ |  |  | ✓ | Ongoing |
| Long-term toxicity testing on invertebrates (preferred species Daphnia) | ✓ |  | ✓ | ✓ | ✓ |  | ✓ | GD 317 published (OECD, 2021a), potential need for further action |
| Reproductive and development toxicity to an additional aquatic invertebrate species | ✓ |  | ✓ |  |  |  | ✓ | Potential need for further action |
| Short-term toxicity to invertebrates | ✓ |  | ✓ | ✓ |  |  | ✓ | Potential need for further action |
| Long-term toxicity testing on invertebrates | ✓ |  | ✓ | ✓ | ✓ |  | ✓ | Potential need for further action |
| Long-term toxicity to sediment organisms | ✓ |  | ✓ | ✓ | ✓ |  | ✓ | Potential need for further action |
| Effects on other, non-target species (flora and fauna) |  |  | ✓ | ✓ |  |  | ✓ | Potential need for further action |
| Additional toxicity studies in several species |  |  | ✓ | ✓ |  |  | ✓ | Potential need for further action |
| Effects on biotic systems – Plants and algae | | | | | | | | |
| Growth inhibition study aquatic plants (algae preferred) | ✓ |  | ✓ | ✓ | ✓ |  | ✓ | Ongoing / GD 317 published (OECD, 2021a) |
| Short-term toxicity to plants | ✓ |  | ✓ | ✓ | ✓ |  | ✓ | Potential need for further action |
| Long-term toxicity testing on plants | ✓ |  | ✓ |  | ✓ |  | ✓ | Potential need for further action |
| If the active substance is to be used in products for action against plants including algae then tests shall be required to assess toxic effects of metabolites from treated plants, if any, where different from those identified in animals |  |  |  | ✓ |  |  |  | Potential need for further action |
| Effects on biotic systems – Microbial toxicity | | | | | | | | |
| Activated sludge respiration inhibition testing | ✓ |  |  | ✓ | ✓ |  |  | Potential need for further action |
| Effects on soil micro-organisms | ✓ |  | ✓ | ✓ | ✓ |  | ✓ | Potential need for further action |
| Effects on biotic systems – Fish toxicity and accumulation | | | | | | | | |
| Short-term toxicity testing on fish: the registrant may consider long-term toxicity testing instead of short-term. | ✓ |  | ✓ | ✓ |  |  | ✓ | Ongoing / GD 317 published (OECD, 2021a) |
| Long-term toxicity testing on fish | ✓ |  | ✓ | ✓ | ✓ |  | ✓ | GD 317 published (OECD, 2021a) |
| Fish early-life stage (FELS) toxicity test | ✓ |  | ✓ | ✓ | ✓ |  | ✓ | GD 317 published (OECD, 2021a) |
| Fish short-term toxicity test on embryo and sac-fry stages | ✓ |  |  | ✓ |  |  |  | GD 317 published (OECD, 2021a) |
| Fish, juvenile growth test | ✓ |  |  | ✓ |  |  |  | GD 317 published (OECD, 2021a) |
| Bioaccumulation in aquatic species, preferably fish | ✓ |  | ✓ | ✓ | ✓ |  | ✓ | Ongoing |
| Effects on biotic systems – Long term toxicity in birds or mammals | | | | | | | | |
| Long-term or reproductive toxicity to birds | ✓ |  | ✓ | ✓ |  |  |  | Potential need for further action |
| Acute oral toxicity to birds and mammals |  |  | ✓ |  |  |  |  | Potential need for further action |
| Toxic effects on livestock and pets |  |  |  | ✓ |  |  |  | Potential need for further action |
| Food and feeding stuffs studies including for food-producing animals and their products (milk, eggs and honey) |  |  |  | ✓ |  |  |  | Potential need for further action |
| Short-term dietary toxicity to birds |  |  | ✓ |  |  |  |  | Potential need for further action |
| Environmental fate and behaviour – (A)biotic degradation | | | | | | | | |
| Degradation | ✓ |  | ✓ | ✓ |  |  |  | Ongoing / Guidance published (OECD, 2018d) |
| Biotic degradation | ✓ |  | ✓ | ✓ |  |  |  | Potential need for further action |
| Ready biodegradability | ✓ |  | ✓ | ✓ | ✓ |  | ✓ | Potential need for further action |
| Simulation testing on ultimate degradation in surface water | ✓ |  | ✓ | ✓ |  |  |  | Potential need for further action |
| Soil simulation testing | ✓ |  | ✓ |  | ✓ |  | ✓ | Potential need for further action |
| Sediment simulation testing | ✓ |  | ✓ | ✓ | ✓ |  | ✓ | Potential need for further action |
| Biological water remediation: aerobic and anaerobic biodegradation; Sewage Treatment Plant simulation test |  |  | ✓ | ✓ | ✓ |  | ✓ | Study report published (OECD, 2021b) / Potential need for further action |
| Biodegradation in marine water |  |  | ✓ | ✓ |  |  | ✓ | Potential need for further action |
| Biodegradation in manure |  |  | ✓ | ✓ |  |  | ✓ | Potential need for further action |
| Abiotic degradation | ✓ |  | ✓ | ✓ |  |  |  | Ongoing |
| Hydrolysis as a function of pH | ✓ |  | ✓ | ✓ |  |  | ✓ | Potential need for further action |
| Identification of degradation products | ✓ |  | ✓ | ✓ | ✓ |  | ✓ | Ongoing |
| Ecotoxicology and environmental fate – Fate and behaviour in the environment | | | | | | | | |
| Fate and behaviour in the environment | ✓ |  | ✓ | ✓ | ✓ |  | ✓ | Potential need for further action |
| Adsorption/desorption screening | ✓ |  | ✓ | ✓ | ✓ |  | ✓ | GD 342 published (OECD, 2021c) |
| Further information on adsorption/desorption | ✓ |  | ✓ | ✓ | ✓ |  | ✓ | GD 342 published (OECD, 2021c) |
| Further information on the environmental fate and behaviour of the substance and/or degradation products | ✓ |  | ✓ | ✓ | ✓ |  | ✓ | Ongoing |

**Table S3**: Overview of EU and RIVM expert opinions on the potential needs that are specific to nanomaterials and broadly relevant (i.e. relevant for multiple regulatory areas). Information requirements (in bold) with similar needs are grouped in single rows and separated by “/”. Expert opinions were summarised to reduce redundancy. Relevant OECD TGs/GDs are those referenced in one or more of the documents used in identifying the regulatory requirements (see SI Table S2) or identified by experts. Listing them here should not be interpreted as a need to adapt all of these documents for nanomaterials but rather as an overview of relevant OECD documents for which the needs for adaptation requires investigation. Current versions of these OECD documents are available online:
[www.oecd.org/science/nanosafety/publications-series-safety-manufactured-nanomaterials.htm](http://www.oecd.org/science/nanosafety/publications-series-safety-manufactured-nanomaterials.htm)
[www.oecd.org/env/ehs/testing/oecdguidelinesforthetestingofchemicals.htm](http://www.oecd.org/env/ehs/testing/oecdguidelinesforthetestingofchemicals.htm)

| Endpoint | Summary of expert opinions on nano specific needs | Relevant OECD TGs/GDs |
| --- | --- | --- |
| Physico-chemical properties | | |
| **Dispersion stability in relevant media**, required for:   - REACH - Cosmetics - Food and feed - Biocides - Medical products - Medical devices | This endpoint is addressed in TG 318 and GD 318 for environmental media. Further action on standardisation is needed for biological media used in toxicology studies relevant for human health, including *in vitro* studies. Such action should include discussion on whether testing requires a stable dispersion in the test or whether testing requires test materials under realistic conditions that may include agglomeration. | TG 318  GD 318 |
| **Stability** (physical and chemical), required for:   - Cosmetics - Food and feed - Biocides - Medical products - Medical devices - Veterinary medical products | This endpoint needs to be addressed in all (eco)toxicity and *in vitro* studies during the exposure period. | - |
| Health effects | | |
| **Reactivity** (catalytic activity, chemical reactivity, photocatalytic activity or radical formation potential), required for:   - Cosmetics - Food and feed - Biocides - Medical devices - Veterinary medical products | For nanomaterials this endpoint is often not measured. Regulations are generally not very specific on methods to be used for these endpoints, although OECD TG 442C (as well as several ISO documents^1^) have been mentioned in this context. There is also a clear link with the oxidising/redox properties.  Generally, the potential of nanomaterials to generate reactive oxygen species (ROS) is measured in both acellular and cellular environments. These ROS measurements can be further optimised and some could be standardised, e.g. Ferric Reduction Ability of Serum (FRAS) assay, 5-(and 6)-Chloromethyl-2’,7’ Dichloro-dihydrofluorescein diacetate (CM-H2DCF-DA) assay (ISO/TS 19006:2016 could be simplified/optimised). In addition, for each protocol there is guidance needed how to control interference. For some materials, specific assays might not be compatible. | TG422C |
| **Cell toxicity (damage to cell/cell membrane, growth, metabolism)**, required for:   - Cosmetics - Food and feed - Medicinal devices - Veterinary medicinal products | OECD GD development may be required for cellular *in vitro* assays in general, addressing issues such as colorimetric interference/media depletion, dosing, sedimentation, exposure periods, target cell selection, etc., as they become increasingly relevant for IATAs/ITSs. Important for a range of regulations including for read-across and grouping. We understand that an overarching *in vitro* TG proposal (non-nanospecific) is currently in development that is expected to go to OECD in the autumn. This, accompanied by improved guidance on sample preparation and dosimetry may be sufficient to address many needs but clearly needs detailed co-ordination.  In the case of *in vitro* tests, it would be necessary to ensure appropriate exposure to the cells through stable suspensions obtained in cell culture medium. It is also necessary to study the interaction / interference with culture medium components. For interpretation of results, also clarity on the number of particles interacting with the cells is needed, and distinction between effects of solute and particle fractions.  There is a need to standardise – a guidance document would probably do, but should such a guidance document maybe also involve other in-vitro issues? There is need for standardised protocols to test cell toxicity. Especially to include guidance on performing the assays while avoiding interference. Also, guidance on which assays to use might be helpful, as usually at least 2 different assays are used that measure a different type of cytotoxicity (e.g. measuring mitochondrial activity and measuring membrane integrity). Also the lactate dehydrogenase (LDH) assay (membrane integrity) could be standardised.  Assays based on mitochondrial activity (MTT, MTS, WST-1, XTT) are widely used and most laboratories have their own specified protocols that could easily be standardised for nanomaterial testing. | - |
| **Inflammation induction (*in vitro)***, required for:   - Cosmetics - Food and feed - Medicinal devices | No standardised method available. Yet, inflammation potential of nanomaterials is considered a central effect of the particles. Many EU project include activities on this, e.g. REFINE ([www.refine-nanomed.eu](file:///C:/Users/berkner/AppData/Local/Microsoft/Windows/INetCache/Content.Outlook/K103RJ1G/www.refine-nanomed.eu)); GRACIOUS ([www.h2020gracious.eu](file:///C:/Users/berkner/AppData/Local/Microsoft/Windows/INetCache/Content.Outlook/K103RJ1G/www.h2020gracious.eu)); SAbyNA ([www.sabyna.eu](file:///C:/Users/berkner/AppData/Local/Microsoft/Windows/INetCache/Content.Outlook/K103RJ1G/www.sabyna.eu)); PATROLS ([www.patrols-h2020.eu](file:///C:/Users/berkner/AppData/Local/Microsoft/Windows/INetCache/Content.Outlook/K103RJ1G/www.patrols-h2020.eu)); SmartNanoTox ([www.smartnanotox.eu](file:///C:/Users/berkner/AppData/Local/Microsoft/Windows/INetCache/Content.Outlook/K103RJ1G/www.smartnanotox.eu)). Not an information requirement as such, but this information may be useful for grouping of nanoforms and Weight-of-Evidence approaches. |  |
| Mutagenicity**: *in vitro* cytogenicity study in mammalian cells or in vitro micronucleus study / In case of positive results *in vitro*, *in vivo* genotoxicity study (somatic and potentially germ cell)**, required for:   - All regulatory areas | OECD TG 487 requires nanospecific adaptations. Applicability of existing OECD TG 474, OECD TG 475, OECD TG 488, and OECD TG 489 is uncertain. Focus needed on whether the particles reach the cells, are taken up by the cells and if they can reach the nucleus. There are initiatives at the HESI Genetic Toxicology Technical Committee ([hesiglobal.org/genetic-toxicology-gttc](https://hesiglobal.org/genetic-toxicology-gttc/)) to develop a protocol for genetic toxicity testing of nanomaterials. | TG 475  TG 483  TG 486  TG 487  TG 488  TG 489 |
| **Acute toxicity (oral / inhalation / dermal route),** required for:   - REACH - Food and feed - Biocides - Medicinal devices - Veterinary medicinal products | The respective OECD TGs for inhalation (OECD TG 403, OECD TG 433, OECD TG 436) have not been adapted for nanomaterials, apart from the fact that the revised OECD GD 39 discussed the pros and cons of the different TG protocols. Therefore, it is a highly relevant research need to investigate and adapt the protocols, with regard to dosing, administration, toxicity criteria, and 3R (replacement, reduction, refinement) compliance where GD39 is not adequate. | TG 402  TG 403  TG 420  TG 423  TG 425  TG 427  TG 433  TG 436  GD 39 |
| **Phototoxicity**, required for:   - Cosmetics - Biocides - Medicinal products | Phototoxicity and photogenotoxicity need further action and TG update to accommodate to nanomaterials | TG 432 |
| Effects on biotic systems | | |
| Effects on terrestrial organism**: Short-term toxicity to invertebrates / Effects on soil micro-organisms / Short-term toxicity to plants / Long-term toxicity testing on invertebrates / Long-term toxicity testing on plants,** required for:   - REACH - Biocides - Food and feed - Medicinal products - Veterinary medicinal products | Much of the focus so far has been towards the aquatic environment. However, soils are a major sink for nanomaterials. Therefore, adaptations for soils testing of nanomaterials needs to be further examined. Most issues with soil testing are likely related to dosing and determining actual exposures. An overarching document for soil testing similar to GD 317 may be required. | TG 207  TG 216  TG 218  TG 219  TG 220  TG 222  TG 225  TG 226  TG 232  TG 233  TG 238  TG 239  TG 241  TG 315  TG 317 |
| Environmental fate and behaviour | | |
| **Biotic degradation / Ready biodegradability / Simulation testing on ultimate degradation in surface water / Soil simulation testing / Biodegradation in manure / Sediment simulation testing**, required for:   - REACH - Biocides - Food and feed - Medicinal products - Veterinary medicinal product | Nano only refers to form, and so nanomaterials may be composed of organic, inorganic and organometallic components. Further, nanomaterials may be organically coated, meaning that regardless of the core (e.g. inorganic) the surface treatment can biodegrade. Therefore, there may be a need to adapt/develop TGs/GD to provide information on the biotic degradation for (parts of) nanomaterials. | TG 301  TG 302B  TG 302C  TG 307  TG 308  TG 309  TG 310  TG 311 |
| **Biological water remediation: aerobic and anaerobic biodegradation; Sewage treatment works simulation test / Biodegradation in marine water**, required for:   - Biocides - Food and feed - Medicinal products - Veterinary medicinal products | Unclear whether methods are applicable for nanomaterials. A biodegradation/removal in Sewage Treatment Plant method (OECD, 2021b) has only been tested for one material. More research on validity of available methodology/TGs is required. Or to harmonise these with the removal in Sewage Treatment Plant method. | TG 303  TG 209  TG 306  TG 318  GD 318 |

1. Reactivity relevant ISO documents include ISO/TS 18827:2017, ISO/TS 19006:2016, ISO 20814:2019

**Table S4**: Overview of EU and RIVM expert opinions on regulatory requirements for which the specific need for further work it remains unclear and requires further investigation. Expert opinions were summarised to reduce redundancy. Information requirements (in bold) with similar needs are grouped in single rows and separated by “/”.

| Endpoint | Nano specific issue/need | Relevant OECD TGs/GDs^1^ |
| --- | --- | --- |
| Physico-chemical properties | | |
| **Oxidising properties**, required for:   - REACH - Cosmetics - Food and feed - Biocides | Test methods for oxidising properties are not applicable to nanomaterials, but their potential contribution to combustion of another material is relevant for the hazard assessment. |  |
| **Relative** **density**, required for:   - REACH - Cosmetics - Food and feed - Biocides - Medical devices - Veterinary medical products | The current guideline may not be sufficient for nanomaterial inhalation studies, effective density may be more relevant. An ISO standard is available for skeletal density (ISO 12154:2014). | TG 109 |
| Health effects | | |
| Skin/eye irritation/damage**: Skin corrosion/irritation (*in vivo*) / Skin corrosion (*in vitro*) / Skin irritation *(in vitro*) / Serious eye damage/eye irritation (*in vivo*) / Serious eye damage/eye irritation (*in vitro*)**, required for:   - All regulatory areas | Skin corrosive materials are often liquids, i.e. not nanomaterials. Applicability for nanomaterials of available OECD or ISO documents is uncertain, it has not been investigated. Potential issues are likely related to dispersibility and dosing. | TG 404  TG 405  TG 430  TG 431  TG 437  TG 438  TG 439  TG 435  TG 460  TG 491  TG 492  TG 494  TG 496 |
| **Skin sensitisation (*in vitro*/*in chemico*) / Skin sensitisation (*in vivo*),** required for:   - REACH - Cosmetics - Biocides - Medicinal products - Medicinal devices - Veterinary medicinal products | Not clear to what extent nanospecific issues are anticipated, if any. These may be covered by overarching guidance documents, e.g. Guidance on Sample Preparation and Dosimetry, or on dispersion. Applicability of nanomaterials to the in vitro skin-sensitisation test methods is under investigation (OECD TG 442D, OECD TG 442E). Perhaps such information could be used to clarify the extent of anticipated nanospecific issues | TG 442A  TG 442B  TG 442C  TG 442D  TG 442E  TG 406  TG 429 |
| **Dermal absorption (*in vitro*)**, required for:   - Cosmetics - Food and feed - Biocides - Medicinal products | Not clear to what extent methods are useful for nanomaterials. EUON report: A critical review of the factors determining dermal absorption of nanomaterials and available tools for the assessment of dermal absorption (doi: [10.2823/97626](https://doi.org/10.2823/97626)) appears a good starting point to see what is needed. However, it is based on a limited number of quality studies and it may be premature to draw a very general conclusion. Measurement of nanomaterials in biological tissues is thus a likely issue here (see also WNT Project 1.10). Further, there are a number of studies available tracing nanomaterials in skin by sophisticated high-resolution analytical tools. A systematic evaluation of the appropriateness of such methods could result in a guidance document, e.g. supporting the nano-specific TG for toxicokinetics under development. | TG 428 |
| **Reproductive toxicity** (includes four information requirements, see Table S2 for details), required for:   - All regulatory areas | Nanomaterials may pass through the placenta. Thus information on the reproduction toxicity, fertility/developmental effects is considered relevant. As for other TGs clear guidance on dispersion is important, as well as measurement of nanomaterials in biological tissues (see also WNT Project 1.10). | TG 414  TG 415  TG 416  TG 421  TG 422  TG 443 |
| **Endocrine disruption**, required for:   - Cosmetics - Food and feed - Biocides | Potential endocrine disruption properties of nanomaterials may potentially be related to the particle properties or to properties of (released) chemical components of a nanomaterial (e.g. a coating). It is unclear which of the properties are most relevant. | TG 230  TG 231  TG 234  TG 440  TG 441  TG 455  TG 456  TG 493  TG 455  TG 456  TG 458  TG 493  GD 150 |
| **Neurotoxicity**, required for:   - Food and feed - Biocides - Medicinal products - Veterinary medicinal products - (REACH^2^) | It is not clear whether TGs are applicable to nanomaterials. The information should also include developmental neurotoxicity. There may be issues with dispersion stability and dosing. | TG 418  TG 419  TG 424  TG 426 |
| **Immunotoxicity,** required for::   - Food and feed - Biocides - Medicinal products - Medicinal devices - Veterinary products - (REACH^2^) | Even for conventional chemicals guidance for immunotoxicity is not available. However, the more AOP key events related to interfering with the immune system will be identified, the more relevant becomes guidance on how to test this. [ASTM E2525-08(2013)](http://www.astm.org/Standards/E2525.htm) is available for nanomaterials. | - |
| Effects on biotic systems | | |
| **Activated sludge respiration inhibition testing,** required for:   - REACH - Biocides - Medicinal products | There may be a need for adaptation of or additional guidance for OECD TG 209. | TG 209  TG 224 |
| **Long-term toxicity testing on aquatic invertebrates (preferred species Daphnia) / Reproductive and development toxicity to an additional aquatic invertebrate species**, required for:   - REACH - Food and feed - Biocides - Medicinal products - Veterinary medicinal products | Applicability issues for long-term testing of aquatic species are related to feeding (interaction/interference of ENM with food); OECD GD 317 already highlights issues with feeding, although some more action may be needed, e.g. on flow-through systems; it appears not feasible to add a whole range of appendices to OECD GD 317. | TG 211  GD 317 |
| **Long-term toxicity to sediment organisms**, required for:   - REACH - Biocides - Food and feed - Human medicinal products - Veterinary medicinal products | OECD GD 317 may be sufficient, but there may be further needs for long-term toxicity testing to sediment organisms | TG 218  TG 219  TG 225  TG 233  TG 238  TG 239 |
| **Long-term or reproductive toxicity to birds / Acute oral toxicity to birds and mammals / Short-term dietary toxicity to birds / Toxic effects on livestock and pets / Food and feeding stuffs studies including for food-producing animals and their products (milk, eggs and honey) / Effects on other, non-target species (flora and fauna),** required for:   - REACH - Biocides - Food and feed - Veterinary medicinal products | Guidance on human health endpoints may apply with regard to dosing. | GD 75  TG 205  TG 206  TG 213  TG 214  TG 223  TG 228  TG 237 |
| Environmental fate and behaviour | | |
| Abiotic degradation: **Hydrolysis as a function of pH**, required for:   - REACH - Biocides - Food and feed - Veterinary medicinal products | Unclear whether existing methods are applicable for nanomaterials. | TG 111 |
| **Fate and behaviour in the environment,** required for:   - REACH - Food and feed - Biocides - Veterinary medicinal products | Unclear whether ongoing projects sufficiently address needs for nanomaterials. Much of the focus so far has been towards the aquatic environment. However, soils are a major sink for nanomaterials. Therefore, there is a need to further examine fate and behaviour of nanomaterials in soils.  Further, there is a need to develop an alternative to the Equilibrium Partitioning Method for nanomaterials. This method used log K_OW_ and other physico-chemical properties as triggering values to determine whether additional testing will be required. However, this method is not suitable for non-rapidly dissolving nanomaterials. | - |

1. Relevant OECD TGs/GDs are those referenced in one or more of the documents used in identifying the regulatory requirements (see SI Table S2) or identified by experts. Current versions of these OECD documents are available online:
   [www.oecd.org/science/nanosafety/publications-series-safety-manufactured-nanomaterials.htm](http://www.oecd.org/science/nanosafety/publications-series-safety-manufactured-nanomaterials.htm)
   [www.oecd.org/env/ehs/testing/oecdguidelinesforthetestingofchemicals.htm](http://www.oecd.org/env/ehs/testing/oecdguidelinesforthetestingofchemicals.htm)
2. Note that in effects on the development of nervous and immune system can be measured in extended one-generation tests which is a (conditional) information requirement within REACH, but the decision to include these parameters is done on a case-by-case basis (ECHA, 2016).

**Table S5:** Overview of expert opinions on potential needs that are specific to nanomaterials but not broadly relevant (e.g. relevant to a specific requirement in only one regulatory area or not much relied upon in risk assessment). Expert opinions were summarised to reduce redundancy. Information requirements (in bold) with similar needs are grouped in single rows and separated by “/”.

| Endpoint | Summary of expert opinions on nano specific needs | Relevant OECD TGs/GDs^1^ |
| --- | --- | --- |
| Physico-chemical properties | | |
| **Dissociation constant**, required for:   - REACH - Cosmetics - Biocides - Medical products - Veterinary medicinal products | The dissociation constant may affect size. It is not clear how the results of OECD TG 112 might be impacted by the presence of a colloidal suspension (OECD, 2009), and also surface modification may play a role (Christensen and Larsen, 2013). As this endpoint is generally not relied upon in risk assessment, it is not considered a high priority. | TG 112 |
| **Stability in organic solvents**, required for:   - REACH - Food and feed - Biocides - Medicinal products - Medicinal devices - Veterinary medicinal products | Stability in organic solvents may need further action, but this has no priority as it is not much relied upon in risk assessment. | - |
| **In-use stability**, required for:   - Cosmetics - Biocides - Medicinal products - Medicinal devices - Veterinary medicinal products | For in-use stability (at relevant storage conditions) issues relate to stability of dispersions, dissolution and chemical stability. | - |
| **Interactions with drugs and other active ingredients (**includes three information requirements, see Table S2 for details), required for:   - Medicinal products - Medicinal devices - Veterinary medical products | Drug loading efficiency, distribution/release of any active ingredient are only relevant for medical frameworks. For other areas (and OECD) this currently has a low priority. | - |
| Health effects | | |
| **Bioburden control / Pharmacokinetic parameters / Pharmacodynamical parameters**, required for:   - Medicinal products - Veterinary medicinal products | Lower priority, as it is only relevant for medical frameworks. | - |
| Effects on biotic systems | | |
| **If the active substance is to be used in products for action against plants including algae then tests shall be required to assess toxic effects of metabolites from treated plants, if any, where different from those identified in animals**, required for:   - Biocides | Similar to degradation issues | - |
| **Additional toxicity studies in several species**, required for:   - Biocides - Food and feed - Veterinary medical products | Needs will depend on specific species | GD 53  TG 241  TG 315  TG 317 |

1. Relevant OECD TGs/GDs are those referenced in one or more of the documents used in identifying the regulatory requirements (see SI Table S2) or identified by experts. Current versions of these OECD documents are available online:
   [www.oecd.org/science/nanosafety/publications-series-safety-manufactured-nanomaterials.htm](http://www.oecd.org/science/nanosafety/publications-series-safety-manufactured-nanomaterials.htm)
   [www.oecd.org/env/ehs/testing/oecdguidelinesforthetestingofchemicals.htm](http://www.oecd.org/env/ehs/testing/oecdguidelinesforthetestingofchemicals.htm)

References

Christensen, F. M., Larsen, P. B., Information Requirements for nanomaterials - IRNANO. Danish Environmental Protection Agency, Copenhagen, Denmark, 2013, pp. 100.

EC, 2001. Directive 2001/83/EC of the European Parliament and of the Council of 6 November 2001 on the Community code relating to medicinal products for human use. Off. J. EU. L 311**,** 67-128.

EC, 2006. Regulation (EC) No 1907/2006 of the European Parliament and of the Council of 18 December 2006 concerning the Registration, Evaluation, Authorisation and Restriction of Chemicals (REACH), establishing a European Chemicals Agency, amending Directive 1999/45/EC and repealing Council Regulation (EEC) No 793/93 and Commission Regulation (EC) No 1488/94 as well as Council Directive 76/769/EEC and Commission Directives 91/155/EEC, 93/67/EEC, 93/105/EC and 2000/21/EC. Off. J. EU. L 396**,** 1-849.

EC, 2009. Regulation (EC) No 1223/2009 of the European Parliament and of the Council of 30 November 2009 on cosmetic products (recast). Off. J. EU. L 342**,** 59-209.

EC, 2015. Regulation (EC) 2015/2283 of the European Parliament and of the Council of 25 November 2015 on novel foods, amending Regulation (EU) No 1169/2011 of the European Parliament and of the Council and repealing Regulation (EC) No 258/97 of the European Parliament and of the Council and Commission Regulation (EC) No 1852/2001. Off. J. EU. L 327**,** 1-22.

ECHA, How ECHA identifies the design for the extended one-generation reproductive toxicity study (EOGRTS) under dossier evaluation. Guidance for the implementation of REACH. European Chemicals Agency (ECHA), Helsinki, Finland, 2016.

EFSA FEEDAP Panel, et al., 2019. Guidance on the assessment of the safety of feed additives for the environment. EFSA J. 17.

EFSA Panel on Food Additives Nutrient Sources added to Food, 2012. Guidance for submission for food additive evaluations. EFSA J. 10**,** 2760.

EFSA Scientific Committee, et al., 2021. Guidance on risk assessment of nanomaterials to be applied in the food and feed chain: human and animal health. EFSA J. 19**,** 6768.

EMA, Guideline on repeated dose toxicity (CPMP/SWP/1042/99 Rev 1 Corr). Committee for Human Medicinal Products (CHMP), European Medicines Agency (EMA), London, UK, 2010, pp. 9.

EMA, Guideline on determining the fate of veterinary medicinal products in manure (EMA/CVMP/ERA/430327/2009). Committee for Medicinal Products for Veterinary Use (CVMP), European Medicines Agency (EMA), London, UK, 2011, pp. 11.

EMA, Joint MHLW/EMA reflection paper on the development of block copolymer micelle medicinal products (EMA/CHMP/13099/2013). Committee for Medicinal Products for Human Use (CHMP), European Medicines Agency (EMA), London, UK, 2013a, pp. 18.

EMA, Reflection paper on the data requirements for intravenous liposomal products developed with reference to an innovator liposomal product (EMA/CHMP/806058/2009/Rev. 02). Committee for Medicinal Products for Human Use (CHMP), European Medicines Agency (EMA), London, UK, 2013b, pp. 13.

EMA, VICH GL23: Studies to evaluate the safety of residues of veterinary drugs in human food: genotoxicity testing (EMA/CVMP/VICH/526/2000). Committee for Medicinal Products for Veterinary Use (CVMP), European Medicines Agency (EMA), London, UK, 2014, pp. 7.

EMA, Guideline on non-clinical local tolerance testing of medicinal products (EMA/CHMP/SWP/2145/2000 Rev. 1, Corr. 1). Committee for Human Medicinal Products (CHMP), European Medicines Agency (EMA), London, UK, 2015a, pp. 9.

EMA, ICH guidance S10 on photosafety evaluation of pharmaceuticals - step 5 (EMA/CHMP/ICH/752211/2012). Committee for Human Medicinal Products (CHMP), European Medicines Agency (EMA), London, UK, 2015b, pp. 17.

EMA, Reflection paper on the data requirements for intravenous iron-based nano-colloidal products developed with reference to an innovator medicinal product (EMA/CHMP/SWP/620008/2012). Committee for Medicinal Products for Human Use (CHMP), European Medicines Agency (EMA), London, UK, 2015c, pp. 11.

EMA, Guideline on the chemistry of active substances. Committee for Medicinal Products for Human Use (CHMP), European Medicines Agency (EMA), London, UK, 2016, pp. 16.

EMA, Guideline on the requirements for the chemical and pharmaceutical quality documentation concerning investigational medicinal products in clinical trials. Committee for Medicinal Products for Human Use (CHMP), European Medicines Agency (EMA), London, UK, 2017, pp. 39.

EMA, Guideline on the environmental risk assessment of medicinal products for human use - Draft (EMEA/CHMP/SWP/4447/00 Rev. 1). Committee for Medicinal Products for Human Use (CHMP), European Medicines Agency (EMEA), London, UK, 2018, pp. 48.

EMA, Advice implementing measures under Article 146(2) of Regulation (EU) 2019/6 on veterinary medicinal products – Scientific recommendation on the revision of Annex II to Regulation (EU) 2019/6 on veterinary medicinal products. Committee for Medicinal Products for Veterinary Use (CVMP), European Medicines Agency (EMA), London, UK, 2019, pp. 89.

EMA, ICH S5 (R3) guideline on reproductive toxicology: Detection of toxicity to reproduction for human pharmaceuticals - step 5 (EMA/CHMP/ICH/544278/1998). Committee for Human Medicinal Products (CHMP), European Medicines Agency (EMA), London, UK, 2020, pp. 127.

EMEA, Note for guidance Specifications: Test Procedures and Acceptance Criteria for New Drug Substances and New Drug Products: Chemical Substances (CPMP/ICH/367/96) Committee for Medicinal Products for Human Use (CPMP), European Medicines Agency (EMEA), London, UK, 2000, pp. 32.

EMEA, Note for Guidance on Carcinogenic Potential (CPMP/SWP/2877/00) Committee for Medicinal Products for Human Use (CPMP), European Medicines Agency (EMEA), London, UK, 2002, pp. 8.

EMEA, VICH GL22 Safety studies for veterinary drug residues in human food: reproduction studies (CVMP/VICH/525/2000). Committee for Veterinary Medicinal Products (CVMP), European Medicines Agency (EMA), London, UK, 2004a, pp. 8.

EMEA, VICH GL31 Safety studies for veterinary drug residues in human food: repeat-dose (90) toxicity testing (CVMP/VICH/484/2002). Committee for Veterinary Medicinal Products (CVMP), European Medicines Agency (EMA), London, UK, 2004b, pp. 5.

EMEA, VICH GL37 Safety of veterinary drugs in human food repeat-dose (chronic) toxicity testing (CVMP/VICH/468/2003). Committee for Veterinary Medicinal Products (CVMP), European Medicines Agency (EMA), London, UK, 2004c, pp. 8.

EMEA, VICH GL38: Environmental impact assessment for veterinary medicinal products, phase II guidance (CVMP/VICH/790/03-FINAL). Committee for Medicinal Products for Veterinary Use (CHMP), European Medicines Agency (EMEA), London, UK, 2004d, pp. 39.

EMEA, VICH GL28: Studies to evaluate the safety of veterinary drugs in human: carcinogenicity testing (CVMP/VICH/645/2001 Rev.1). Committee for Medicinal Products for Veterinary Use (CVMP), European Medicines Agency (EMEA), London, UK, 2005, pp. 6.

EMEA, Guideline on the environmental risk assessment of medicinal products for human use (EMEA/CHMP/SWP/4447/00 corr 2) Committee for Medicinal Products for Human Use (CPMP), European Medicines Agency (EMEA), London, UK, 2006a, pp. 12.

EMEA, Guideline on the non-clinical investigation of the dependence potential of medicinal products (EMEA/CHMP/SWP/94227/2004) Committee for Medicinal Products for Human Use (CPMP), European Medicines Agency (EMEA), London, UK, 2006b, pp. 12.

EMEA, Note for guidance on immunotoxicity studies for human pharmaceuticals (CHMP/167235/2004) Committee for Medicinal Products for Human Use (CPMP), European Medicines Agency (EMEA), London, UK, 2006c, pp. 13.

EMEA, Guideline on risk assessment of medicinal products on human reproduction and lactation: from data to labelling (EMEA/CHMP/203927/2005) Committee for Medicinal Products for Human Use (CPMP), European Medicines Agency (EMEA), London, UK, 2008a, pp. 18.

EMEA, Guideline on the need for non-clinical testing in juvenile animals on human pharmaceuticals for paediatric indications (EMEA/CHMP/SWP/169215/2005) Committee for Medicinal Products for Human Use (CPMP), European Medicines Agency (EMEA), London, UK, 2008b, pp. 9.

EMEA, Note for guidance on genotoxicity testing and data interpretation for pharmaceuticals intended for human use (EMA/CHMP/ICH/126642/2008) Committee for Medicinal Products for Human Use (CPMP), European Medicines Agency (EMEA), London, UK, 2008c, pp. 28.

EMEA, VICH GL33 Safety studies for veterinary drug residues in human food: general approach to testing (EMEA/CVMP/VICH/486/02-Rev.2). Committee for Veterinary Medicinal Products (CVMP), European Medicines Agency (EMA), London, UK, 2009, pp. 8.

EU, 2011. Regulation (EU) No 1169/2011 of the European Parliament and of the Council of 25 October 2011 on the provision of food information to consumers, amending Regulations (EC) No 1924/2006 and (EC) No 1925/2006 of the European Parliament and of the Council, and repealing Commission Directive 87/250/EEC, Council Directive 90/496/EEC, Commission Directive 1999/10/EC, Directive 2000/13/EC of the European Parliament and of the Council, Commission Directives 2002/67/EC and 2008/5/EC and Commission Regulation (EC) No 608/2004. Off. J. EU. L 304**,** 18-63.

EU, 2012. Regulation (EU) No 528/2012 of the European Parliament and of the Council of 22 May 2012 concerning the making available on the market and use of biocidal products. Off. J. EU. L 167**,** 1-123.

EU, 2013. Commission Regulation (EU) No 283/2013 efof 1 March 2013 setting out the data requirements for active substances, in accordance with Regulation (EC) No 1107/2009 of the European Parliament and of the Council concerning the placing of plant protection products on the market. Official Journal. L 93**,** 1-84.

EU, 2017. Regulation (EU) 2017/745 of the European Parliament and of the Council of 5 April 2017 on medical devices, amending Directive 2001/83/EC, Regulation (EC) No 178/2002 and Regulation (EC) No 1223/2009 and repealing Council Directives 90/385/EEC and 93/42/EEC. Official Journal of the European Union. L 117**,** 1-175.

EU, 2018. Commission Regulation (EU) 2018/1881 of 3 December 2018 amending Regulation (EC) No 1907/2006 of the European Parliament and of the Council on the Registration, Evaluation, Authorisation and Restriction of Chemicals (REACH) as regards Annexes I, III,VI, VII, VIII, IX, X, XI, and XII to address nanoforms of substances. Off. J. EU. L 308**,** 1-20.

EU, 2019. Regulation (EU) 2019/6 of the European Parliament and of the Council of 11 December 2018 on veterinary medicinal products and repealing Directive 2001/82/EC. Off. J. EU. L 4**,** 43–167.

EU, 2021. Commission Delegated Regulation (EU) 2021/805 of 8 March 2021 amending Annex II to Regulation (EU) 2019/6 of the European Parliament and of the Council. Off. J. EU. L 180**,** 3-77.

ISO, ISO 10993-20:2006 – Biological evaluation of medical devices — Part 20: Principles and methods for immunotoxicology testing of medical devices. International Organization for Standardization (ISO), 2006, pp. 17.

ISO, ISO 10993-5:2009 – Biological evaluation of medical devices — Part 5: Tests for in vitro cytotoxicity. International Organization for Standardization (ISO), 2009, pp. 34.

ISO, ISO 10993-3:2014 – Biological evaluation of medical devices — Part 3: Tests for genotoxicity, carcinogenicity and reproductive toxicity. International Organization for Standardization (ISO), Geneva, Switzerland, 2014, pp. 34.

ISO, ISO 10993-11:2017 – Biological evaluation of medical devices — Part 11: Tests for systemic toxicity. International Organization for Standardization (ISO), Geneva, Switzerland, 2017a, pp. 29.

ISO, ISO/TR 10993-22:2017 – Biological evaluation of medical devices— Part 22: Guidance on nanomaterials. International Organization for Standardization (ISO), 2017b, pp. 68.

ISO, ISO 10993-10:2021 – Biological evaluation of medical devices — Part 10: Tests for skin sensitization. International Organization for Standardization (ISO), Geneva, Switzerland, 2021a, pp. 48.

ISO, ISO 10993-23:2021 – Biological evaluation of medical devices — Part 23: Tests for irritation. International Organization for Standardization (ISO), Geneva, Switzerland, 2021b, pp. 60.

OECD, OECD Series on the Safety of Manufactured Nanomaterials, No. 15. Preliminary Review of OECD Test Guidelines for their Applicability to Manufactured Nanomaterials. Organisation for Economic Co-operation and Development (OECD), Paris, France, 2009.

OECD, OECD Guidelines for the Testing of Chemicals 318: Dispersion Stability of Nanomaterials in Simulated Environmental Media Organisation for Econonomic Co-operation and Development (OECD), Paris, France, 2017.

OECD, OECD Guidelines for Testing of Chemicals 412: Subacute Inhalation Toxicity: 28-Day Study. Organisation for Econonomic Co-operation and Development (OECD), Paris, France, 2018a.

OECD, OECD Guidelines for Testing of Chemicals 413: Subchronic Inhalation Toxicity: 90-day Study. Organisation for Econonomic Co-operation and Development (OECD), Paris, France, 2018b.

OECD, OECD Series on Testing and Assessment, No. 39. Guidance Document on Inhalation Toxicity Studies (Second Edition). Organisation for Economic Co-operation and Development (OECD), Paris, France, 2018c.

OECD, OECD Series on the Safety of Manufactured Nanomaterials, No. 86 - Assessment of Biodurability of Nanomaterials and their Surface ligands. Organisation for Economic Co-operation and Development (OECD), Paris, France, 2018d, pp. 90.

OECD, OECD Series on Testing and Assessment No. 317 - Guidance Document on Aquatic and Sediment Toxicological Testing of Nanomaterials. Organisation for Economic Co-operation and Development (OECD), Paris, France, 2021a.

OECD, OECD Series on Testing and Assessment No. 340 - Study Report on a Test for Removal in Wastewater Treatment Plants of Gold Manufactured nanomaterials (MN): Activated Sludge Sorption Isotherm Organisation for Economic Co-operation and Development (OECD), Paris, France, 2021b.

OECD, OECD Series on Testing and Assessment No. 342 - Guidance Document on Testing Nanomaterials Using OECD TG No. 312 "Leaching in Soil Columns". Organisation for Economic Co-operation and Development (OECD), Paris, France, 2021c.

OECD, OECD Guidelines for the Testing of Chemicals, Test No. 124: Determination of the Volume Specific Surface Area of Manufactured Nanomaterials. Organisation for Economic Co-operation and Development (OECD), Paris, France, 2022a.

OECD, OECD Guidelines for the Testing of Chemicals, Test No. 125: Nanomaterial Particle Size and Size Distribution of Nanomaterials. Organisation for Economic Co-operation and Development (OECD), Paris, France, 2022b.

SCCS, The SCCS notes of guidance for the testing of cosmetic ingredients and their safety evaluation - 10^th^ revision. . Scientific Committee on Consumer Safety (SCCS), Brussels, Belgium, 2018, pp. 152.

SCCS, Guidance on the safety assessment of nanomaterials in cosmetics. Scientific Committee on Consumer Safety (SCCS), Brussels, Belgium, 2019, pp. 113.

SCENIHR, Opinion on Guidance on the Determination of Potential Health Effects of Nanomaterials Used in Medical Devices. Scientific Committee on Emerging and Newly Identified Health Risks (SCENIHR), European Commission Brussels, Belgium, 2015.
